# Anthocyanins from *Hibiscus syriacus* L. Inhibit NLRP3 Inflammasome in BV2 Microglia Cells by Alleviating NF-*κ*B- and ER Stress-Induced Ca^2+^ Accumulation and Mitochondrial ROS Production

**DOI:** 10.1155/2021/1246491

**Published:** 2021-02-04

**Authors:** Ilandarage Menu Neelaka Molagoda, Kyoung Tae Lee, Yung Hyun Choi, Jayasingha Arachchige Chathuranga Chanaka Jayasingha, Gi-Young Kim

**Affiliations:** ^1^Department of Marine Life Science, Jeju National University, Jeju 63243, Republic of Korea; ^2^Forest Biomaterials Research Center, National Institute of Forest Science, Jinju 52817, Republic of Korea; ^3^Department of Biochemistry, College of Oriental Medicine, Dong-Eui University, Busan 47227, Republic of Korea

## Abstract

Anthocyanins from the petals of *Hibiscus syriacus* L. (PS) possess anti-inflammatory, antioxidant, and antimelanogenic activities. However, it remains unclear whether PS inhibit the NLR family pyrin domain-containing 3 (NLRP3) inflammasome activation and assembly. This study is aimed at investigating whether PS downregulate NLRP3-mediated inflammasome by inhibiting nuclear factor-*κ*B (NF-*κ*B) and endoplasmic reticulum (ER) stress. BV2 microglia cells were treated with PS in the presence of lipopolysaccharide and adenosine triphosphate (LPS/ATP), and the NLRP3-related signaling pathway was investigated. In this study, we found that LPS/ATP treatment activated the NLRP3 inflammasome, which resulted in the release of interleukin-1*β* (IL-1*β*) and IL-18. Meanwhile, PS reduced LPS/ATP-mediated NLRP3 inflammasome at 12 h by inhibiting ER stress-mediated Ca^2+^ accumulation and subsequent mitochondrial reactive oxygen species (mtROS) production, which, in turn, decreased IL-1*β* and IL-18 release. Furthermore, PS inhibited the NLRP3 inflammasome 1 h after LPS/ATP treatment by suppressing the NF-*κ*B pathway, which downregulated Ca^2+^ accumulation and mtROS production. These data showed that PS negatively regulated activation of the NLRP3 inflammasome in a time-different manner by inhibiting the NF-*κ*B signaling pathway in the early stage and the ER stress response in the late stage. The pathways shared Ca^2+^ accumulation-mediated mtROS production, which was significantly inhibited in the presence of PS. In conclusion, our results suggested that PS has potential as a supplement against NLRP3 inflammasome-related inflammatory disorders; nevertheless, further studies are needed to determine the effect of PS in the noncanonical NLRP3 inflammasome pathways and pathological conditions in vivo.

## 1. Introduction

Inflammasomes are a group of multiprotein cytosolic receptors that sense pathogen- and danger-associated molecular patterns in response to pathogen infection and cellular damages and thereby trigger the activation of caspase-1 and consequent maturation of inflammatory cytokines such as interleukin-1*β* (IL-1*β*) and IL-18 [1]. Among the inflammasomes, NATCH, LRR, and PYD domain-containing protein 3 (NLRP3) is well characterized [2] and is associated with several human disorders such as Alzheimer's disease, obesity, rheumatoid arthritis, asthma, nonalcoholic fatty liver disease, and autoimmune encephalitis [3]. Furthermore, the activation of NLRP3 is also associated with the pathogenesis of cardiovascular diseases including atherosclerosis, hypertension, infectious cardiac diseases, and heart failures [4]. The initiation and activation of the NLRP3 inflammasome are associated with two main signals; the first is induced by microbial molecules, such as bacterial lipopolysaccharide (LPS) or endogenous cytokines, and leads to the upregulation of *NLRP3* synthesis, as well as *IL-1β* and *IL-18* through the activation of NF-*κ*B; the second is induced by a plethora of stimuli such as extracellular ATP, viral RNA, and particulate matter and facilitates the arrangement of inflammasome components [5]. When the structurally distinct molecules trigger the recruitment of apoptosis-associated speck-like proteins containing CARD (ASC) and pro-caspase-1 to NLRP3, the complex subsequently cleaved pro-caspase-1 to several active caspase-1, including p20 and p10 [6]. Active caspase-1 finally cleaves IL-1*β* and IL-18 precursors to their actively mature form, thereby initiating caspase-1-mediated inflammation and cell death [7].

Proteins in the endoplasmic reticulum (ER) are properly folded and assembled with assistance from ER chaperones. When proteins are incorrectly folded, they are disposed through the proteasome-mediated degradation pathway, which is known as ER-associated protein degradation (ERAD) [8]. However, when unfolded proteins or nascent proteins accumulate and exceed the folding capacity of the ER, mammalian cells activate a defense mechanism, called ER stress [9]. During ER stress, the induction of chaperones and the ERAD components, the translational attenuation of proteins, and the induction of apoptosis are progressively generated [10]. Immunoglobulin protein (BiP), also known as GRP78, belongs to the heat shock protein family and is a highly abundant ER chaperone that resides in the ER lumen [11]. Under normal physiological conditions, GRP78 remains intact with three unfolded protein response (UPR) transmembrane sensor proteins: activating transcription factor 6 (ATF6), double-stranded RNA-activated protein kinase-like endoplasmic reticulum kinase (PERK), and inositol-requiring kinase 1 (IRE1). Under ER stress conditions, GRP78 is released from the UPR sensors to facilitate the unfolded protein response [12]. In particular, the GRP78-PERK signaling pathway has been extensively studied in the context of ER stress [13, 14]. PERK is released from GRP78 and subsequently phosphorylates eukaryotic translation initiation factor 2*α* (eIF2*α*), resulting in the attenuation of protein translation. Thus, eIF2*α* is allowed to interact with the coding region of ATF4 and ultimately results in the upregulation of CCAAT-enhancer-binding protein homologous protein (CHOP) [15], which induces ER stress-mediated cell death [16].

NLRP3 resides in the ER prior to its activation, and during ER stress, it is associated with the formation of an inflammasome complex in the cytosol [17]. IRE1*α*-mediated PERK inhibition reduces caspase-1 activation and IL-1*β* maturation through suppression of the NLRP3 inflammasome, indicating that ER stress is involved in the activation of the NLRP3 inflammasome [18]. Interestingly, Ca^2+^ mobilization is also implicated in the activation of the NLRP3 inflammasome in several cell lines [19]. In ER stress conditions, Ca^2+^ is released from the ER lumen, which then enters the mitochondria through voltage-dependent anion-selective channels and the mitochondrial calcium uniporter [20]. Excessive accumulation of Ca^2+^ in the mitochondrial matrix subsequently creates mitochondrial depolarization and mitochondrial reactive oxygen species (mtROS) production [21]. Finally, mtROS enhances the assembly of the NLRP3 inflammasome complex and thereby promotes the maturation of IL-1*β* and IL-18 by activating caspase-1 [5].

*Hibiscus syriacus* L. is widely distributed throughout Southern and Northern Asia. Several dried plant parts of *H. syriacus* L. have been used as an antidote or antipyretic in Korean traditional remedies [22]. Recently, we found that anthocyanins from dried flower petals of *H. syriacus* L. (PS) exhibited anti-inflammatory [23], antioxidative [24], and antimelanogenic [25] properties. However, it remains unclear whether PS inhibit NLRP3 inflammasome-mediated inflammation. In this study, we found that PS were a potential inhibitor of the NLRP3 inflammasome and, in turn, suppressed the maturation of IL-1*β* and IL-18 through the inhibition of NF-*κ*B- and ER stress-mediated Ca^2+^ mobilization and mtROS production, which led to the protection of LPS/ATP-induced cell death in BV2 microglia cells.

## 2. Materials and Methods

### 2.1. Reagents and Antibody

LPS from *Escherichia coli* O111:B4, ATP, salubrinal, 3-(4,5-dimethylthiazol-2-yl)-2,5-diphenyl-tetrazolium bromide (MTT), and Fluo-4 AM were obtained from Sigma (St. Louis, MO, USA). Dulbecco's modified Eagle's medium (DMEM), fetal bovine serum (FBS), and antibiotic mixture were obtained from WelGENE (Gyeongsan-si, Gyeongsangbuk-do, Republic of Korea). Antibodies against ASC (sc-514414, 22 kDa), pro-caspase-1 (sc-56036, 45 kDa), p50 (sc-8414, 50 kDa), p65 (sc-8008, 65 kDa), CHOP (sc-7351, 30 kDa), nucleolin (sc-8031, 110 kDa), *β*-actin (sc-69879, 43 kDa), and peroxidase-labeled anti-mouse immunoglobulins (sc-16102) were purchased from Santa Cruz Biotechnology (Santa Cruz, CA, USA). Antibodies against eIF2*α* (PA5-27366, 35 kDa), phospho- (p-) eIF2*α* (MA5-15133, 35 kDa), and ATF4 (PA5-19521, 48 kDa) were purchased from Thermo Fisher Scientific (San Jose, CA, USA). Antibody against NLRP3 (15101S, 110 kDa) was purchased from Cell Signaling Technology (Danvers, MA, USA). Peroxidase-labeled anti-rabbit immunoglobulin (KO211708) was obtained from Koma Biotechnology (Seoul, Republic of Korea). Alexa Fluor 488-conjugated secondary antibody and Dako Faramount Aqueous Mounting Media were purchased from Abcam (Cambridge, MA, UK) and Dako (Carpinteria, CA, USA), respectively. PS were purified in our previous study, and purity was approximately 95% [25]. All other chemicals were purchased from Sigma.

### 2.2. Cell Culture and Viability Assay

BV2 microglia cells (from E.H. Joe, Ajou University School of Medicine, Suwon-si, Gyeonggi-do, Republic of Korea) were cultured in DMEM supplemented with 5% FBS. The cells (1 × 10^5^ cells/mL) were treated with the indicated concentrations of PS (0–1000 *μ*g/mL) for 24 h, and then, MTT assay was performed. In a parallel experiment, cell images were captured by phase contrast microscopy (Ezscope i900PH, Macrotech, Goyang-si, Gyeonggi-do, Republic of Korea).

### 2.3. Analysis of Viability and Dead Cell Population

BV2 microglia cells were treated with the indicated concentrations of PS (0–1000 *μ*g/mL) for 24 h. In a parallel experiment, the cells were pretreated with PS for 2 h and then incubated with 1 *μ*g/mL LPS 2 h before treatment with 1 mM ATP in the presence of LPS (LPS/ATP) for another 24 h. Then, the harvested cells were washed with ice-cold phosphate-buffered saline (PBS) and stained with Muse Count & Viability Kit (MCH100102, Luminex Corp., Austin, TX, USA) for 5 min. Viability and dead cell populations were measured by a Muse Cell Analyzer (Luminex Corp.).

### 2.4. Reverse Transcription Polymerase Chain Reactions (RT-PCR)

BV2 microglia cells were pretreated with PS for 2 h and then incubated with LPS/ATP. Total RNA was extracted using an Easy-BLUE RNA Extraction Kit (iNtRON Biotechnology, Seongnam-si, Gyeonggi-do, Republic of Korea) according to the manufacturer's instructions. The genes of interest were amplified from cDNA using the One-Step RT-PCR Premix (iNtRON Biotechnology). All PCR were performed in the same conditions of denaturation at 94°C for 30 s and extension at 72°C for 30 s. Glyceraldehyde 3-phosphate dehydrogenase (*GAPDH*) was used as a loading control to evaluate the relative expression of *NLRP3*, *ASC*, *IL-1β*, and *IL-18*. The specific primers and PCR conditions used in this experiment are listed (Table 1) [25].

### 2.5. Western Blotting

BV2 microglia cells were pretreated with PS for 2 h and then stimulated with LPS/ATP. Total protein was prepared using Protein Lysis Buffer (iNtRON Biotechnology) at the indicated time points. Lysates were centrifuged at 14,000 g at 4°C for 20 min, and supernatants were collected. In a parallel experiment, the cells were pretreated with PS for 2 h and then incubated with LPS/ATP for 1 h. Nuclear protein was extracted using NE-PER Nuclear and Cytoplasmic Extraction Reagents (Pierce, Rockford, IL, USA). Protein concentrations were measured using a Bio-Rad Protein Assay Kit (Bio-Rad, Hercules, CA, USA), and western blotting was performed. The images were captured by ImageQuant LAS 500 (GE Healthcare Bio-Sciences AB, Uppsala, Sweden). The expressional values of each protein were normalized to *β*-actin or nucleolin.

### 2.6. Measurement of IL-1*β* and IL-18 by Enzyme-Linked Immunosorbent Assay (ELISA)

BV2 microglia cells were treated with PS (0–400 *μ*g/mL) for 2 h and then incubated with LPS/ATP for 48 h. The supernatants were assayed for concentrations of IL-1*β* and IL-18 using ELISA kits (Thermo Fisher Scientific) according to the manufacturer's protocol.

### 2.7. Immunofluorescence of p65

BV2 microglia cells (1 × 10^4^ cells/mL) were seeded on 3% gelatin-coated coverslips, and PS (400 *μ*g/mL) was treated 2 h before LPS/ATP treatment for 1 h. The cells were fixed with 4% paraformaldehyde for 10 min at 37°C and permeabilized with 0.1% Triton X-100 for 10 min at room temperature. Then, the cells were blocked with 10% donkey serum and incubated with p65 antibody (1 : 100 in 10% donkey serum) at 4°C. Alexa Fluor 488-conjugated secondary antibody was treated for 2 h, and the cells were counterstained with nuclear staining dye, DAPI (300 nM). The coverslips were mounted onto glass slides with Dako Faramount Aqueous Mounting Media, and fluorescence images were captured by CELENA S Digital Imaging System (Logos Biosystems, Anyang-si, Gyeonggi-do, Republic of Korea).

### 2.8. Cytosolic Ca^2+^ Staining

BV2 microglia cells were treated with 400 *μ*g/mL PS 2 h before treatment with LPS/ATP for another 1 h and 12 h. The cells were washed with PBS, and intracellular Ca^2+^ was stained by 1 *μ*M Fluo-4 AM. Cell images were captured by CELENA S Digital Imaging System.

### 2.9. mtROS Staining

BV2 microglia cells were treated with 400 *μ*g/mL PS for 2 h and then stimulated with LPS/ATP for another 1 h and 12 h. The cells were incubated with 0.5 *μ*M MitoTracker Green (Thermo Fisher Scientific) for 30 min and stained with 2 *μ*M MitoSOX Red (Thermo Fisher Scientific) for 10 min. Cell images were captured by CELENA S Digital Imaging System.

### 2.10. Mitochondrial Depolarization

BV2 microglia cells were treated with the indicated concentrations of PS (0–400 *μ*g/mL) for 2 h. After stimulating with LPS/ATP for 12 h, the cells were washed with PBS and incubated with a Muse MitoPotential Kit (MCH100110, Luminex Corp.) for 30 min. Mitochondrial membrane depolarization was measured using a Muse Cell Analyzer.

### 2.11. Transient Knockdown of CHOP and NLRP3

BV2 microglia cells were transfected with small interfering RNA (siRNA) of *CHOP* (siCHOP, sc-35438, Santa Cruz Biotechnology) and *NLRP3* (siNLRP3, sc-45470, Santa Cruz Biotechnology) using G-Fectin (Genolution Pharmaceuticals Inc., Seoul, Republic of Korea) for 15 min. The transfection mixture was added to the cells 48 h before LPS/ATP treatment. Transfection efficiency was evaluated by western blotting.

### 2.12. Statistical Analysis

All RT-PCR bands and western blots were quantified by ImageJ 1.50i (National Institutes of Health, Bethesda, VA, USA) and then statistically analyzed by Sigma plot 12.0. All data represented the mean of at least three independent experiments. Significant differences between groups were determined using a Student *t-*test and an unpaired one-way ANOVA with Bonferroni correction.

## 3. Results

### 3.1. PS Protect BV2 Microglia Cells from LPS/ATP-Induced Cell Death

**Figure 1:**
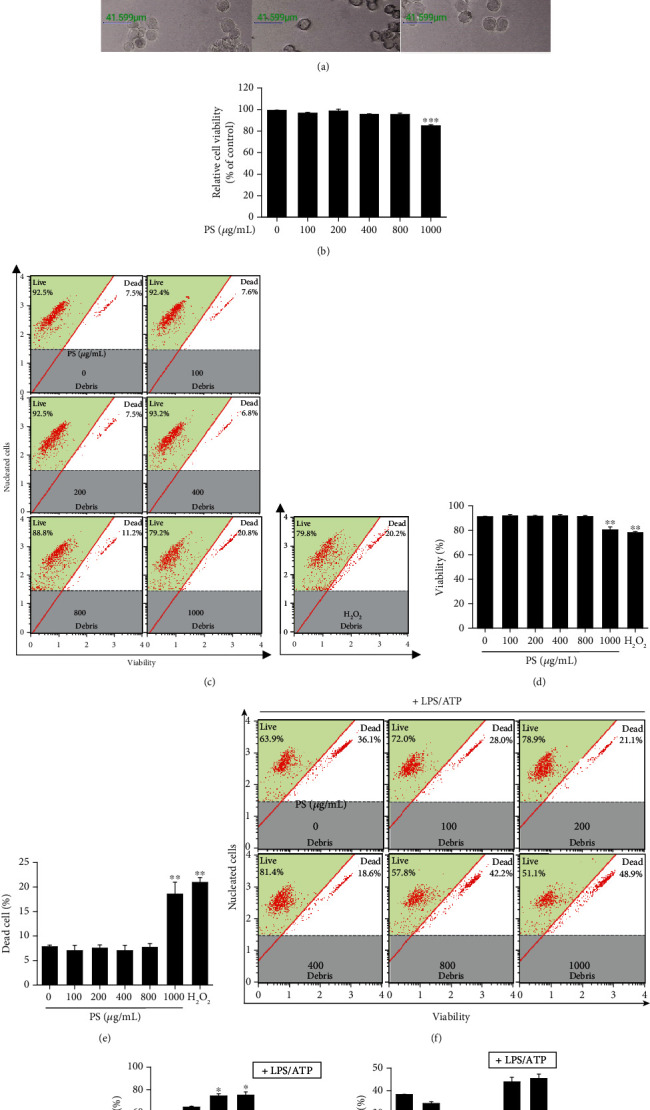
PS protect BV2 microglia cells from LPS/ATP-induced cell death. BV2 microglia cells were treated with the indicated concentrations (0–1000 *μ*g/mL) of PS for 24 h. (a) Cell morphology was observed using phase contrast microscopy (×10). Scale bars = 40 *μ*m. (b) Relative cell viability was measured using an MTT assay. (c) Cell viability and dead cell population were measured using a Muse Cell Viability Kit. Hydrogen peroxide (H_2_O_2_, 100 *μ*M) was used as a positive control. Percentage of (d) viable and (e) dead cells was shown. (f–h) In a parallel experiment, the cells were pretreated with the indicated concentrations of PS (0–1000 *μ*g/mL) for 2 h. After LPS/ATP treatment for 24 h, (f) viable and dead cell populations were measured using a Muse Cell Viability Kit. Percentage of (g) viable and (h) dead cells was shown. ^∗∗∗^*p* < 0.001 and ^∗∗^*p* < 0.01 vs. untreated cells; ^∗^*p* < 0.05 vs. LPS/ATP-treated cells.

To evaluate the effect of PS in cell viability, we treated BV2 microglia cells with the indicated concentrations of PS (0–1000 *μ*g/mL) for 24 h. We found that PS did not result in a significant change in cell morphology (Figure 1(a)). Relative cell viability data showed that below 800 *μ*g/mL showed no cytotoxicity; however, the highest concentration of fisetin (1000 *μ*g/mL) was accompanied by a significant reduction in MTT activity (85.7% ± 1.1%) compared with that in the untreated cells (Figure 1(b)). Furthermore, evaluation of viability and dead cell population was performed using flow cytometry (Figure 1(c)). The data showed that 1000 *μ*g/mL PS significantly decreased cell viability to 81.3% ± 2.4% (Figure 1(d)) and was associated with a significant increase in dead cell population to 18.7% ± 2.4% (Figure 1(e)). No cytotoxicity and cell death populations were observed at PS concentrations below 800 *μ*g/mL. The cytoprotective effect of PS was also assessed in the LPS/ATP-treated cells using flow cytometry (Figure 1(f)). As expected, LPS/ATP-treated cells had significantly reduced cell viability at 61.6% ± 0.2% (Figure 1(g)) and the dead cell population was 38.4% ± 0.2% (Figure 1(h)). Interestingly, pretreatment with PS increased the cell viability to 75.3% ± 1.9% and 76.2% ± 2.8% (Figure 1(g)) and decreased cell death to 23.7% ± 1.9% and 21.8% ± 2.8% (Figure 1(f)) at 200 and 400 *μ*g/mL, respectively. However, no effect on LPS/ATP-induced cell viability and cell death populations was observed with 800 and 1000 *μ*g/mL PS. Therefore, 400 *μ*g/mL PS was used as the maximum concentration in this study. Collectively, these results indicated that low concentrations of PS protect BV2 microglia cells from LPS/ATP-induced cell death.

### 3.2. PS Downregulate the LPS/ATP-Induced NLRP3 Inflammasome and Consequently Inhibit the Release of IL-1*β* and IL-18

**Figure 2:**
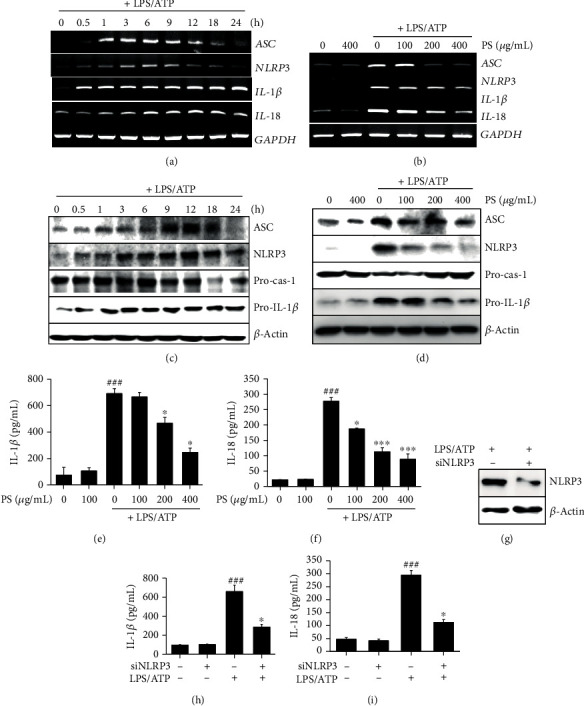
PS inhibit NLRP3 inflammasome-induced IL-1*β* and IL-18 release. (a) BV2 microglia cells were stimulated with LPS/ATP. Total mRNA was isolated at the indicated time points, and RT-PCR was performed. (b) The indicated concentrations of PS (0–400 *μ*g/mL) were pretreated for 2 h prior to stimulation with LPS/ATP. Total mRNA was isolated at 6 h, and RT-PCR was performed. *GAPDH* was used as the loading control. (c) In a parallel experiment, total proteins were isolated at the indicated time points after LPS/ATP treatment, and western blotting was performed. (d) Total proteins were also isolated 12 h after LPS/ATP treatment, and western blotting was performed. Cell culture medium was collected at 48 h, and ELISA was performed to quantify the concentrations of (e) IL-1*β* and (f) IL-18. (g–i) In a parallel experiment, BV2 microglia cells were transfected with siNLRP3 for 48 h and stimulated with LPS/ATP. (g) Transfection efficiency was confirmed by western blotting. Cell culture medium was collected at 48 h, and ELISA was performed to quantify the concentrations of (h) IL-1*β* and (i) IL-18. ^###^*p* < 0.001 vs. untreated cells; ^∗∗∗^*p* < 0.001 and ^∗^*p* < 0.05 vs. LPS/ATP-treated cells.

To address whether PS downregulate the LPS/ATP-induced NLRP3 inflammasome, total mRNA was isolated at the indicated time points, and RT-PCR was performed to check the expression of *NLRP3*, *ASC*, *IL-1β*, and *IL-18*. *NLRP3* and *ASC* were expressed from 0.5 h in response to LPS/ATP (Figure 2(a)). The maximal expression of both *NLRP3* and *ASC* was observed 6 h after LPS/ATP treatment, and the expression gradually decreased from 9 h. We also found that both *IL-1β* and *IL-18* were expressed from 0.5 h after LPS/ATP and sustained for 24 h. Then, we verified whether PS inhibited the gene expression 6 h after LPS/ATP treatment. As expected, PS gradually downregulated all genes tested in this study in a concentration-dependent manner (Figure 2(b)). In addition, as shown in Figure 2(c), inflammasome components such as ASC and NLRP3 were slightly expressed from 3 h and reached maximal levels at 12 h after LPS/ATP treatment, accompanied by decreased expression of pro-caspase-1 from 18 h. Aligned with the RT-PCR data, pro-IL-1*β* was expressed from 1 h after LPS/ATP treatment and sustained by 24 h (Figure 1(c)). However, PS markedly downregulated the expression of ASC, NLRP3, and pro-IL-1*β* 12 h after LPS/ATP treatment, and the reduced level of pro-caspase-1 was restored by the presence of PS (Figure 2(d)). Additionally, LPS/ATP treatment resulted in a significant release of IL-1*β* (695.8 ± 38.4 pg/mL) and IL-18 (279.9 ± 12.3 pg/mL) at 48 h; however, PS significantly downregulated the release in a concentration-dependent manner (671.1 ± 33.4, 472.1 ± 44.3, and 251.9 ± 31.8 pg/mL IL-1*β* and 189.6 ± 2.9, 115.1 ± 13.4, and 90.8 ± 17.7 pg/mL IL-18 at 100, 200, and 400 *μ*g/mL, respectively). Finally, the cells were transfected with siNLRP3 to confirm the significance of NLRP3 inflammasome formation in IL-1*β* and IL-18 release. As expected, silencing of *NLRP3* downregulated its expression (Figure 2(g)) and resulted in the inhibition of LPS/ATP-induced IL-1*β* (658.1 ± 67.8 pg/mL) and IL-18 (294.8 ± 18.1 pg/mL) release to 285.6 ± 28.9 pg/mL (Figure 2(h)) and 112.2 ± 10.6 pg/mL (Figure 2(i)), respectively, indicating that NLRP3 inflammasome plays a vital role in IL-1*β* and IL-18 release. These results indicate that PS downregulate LPS/ATP-induced NLRP3 inflammation and in turn inhibit the release of IL-1*β* and IL-18.

### 3.3. PS Inhibit ER Stress-Induced Ca^2+^ Accumulation, Which Then Downregulates the NLRP3 Inflammasome

**Figure 3:**
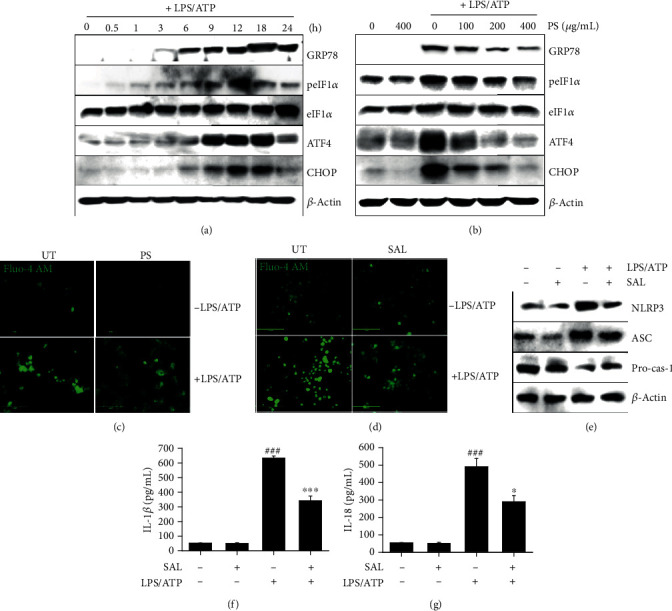
PS inhibit the LPS/ATP-induced NLRP3 inflammasome by downregulating ER stress-induced Ca^2+^ accumulation. (a) BV2 microglia cells were stimulated with LPS/ATP. Total proteins were isolated, and western blotting was performed. (b) In a parallel experiment, the cells were pretreated with the indicated concentrations of PS (0–400 *μ*g/mL) for 2 h prior to stimulation with LPS/ATP for 12 h. Total protein was extracted, and western blotting was performed. BV2 microglia cells were pretreated with (c) PS (400 *μ*g/mL) and (d) salubrinal (SAL, 10 *μ*M) for 2 h prior to stimulation with LPS/ATP for 12 h. The cells were stained with 1 *μ*M Fluo-4 AM, and cell images were captured using CELENA S Digital Imaging System. (e) Under SAL-treated conditions, the total protein was isolated at 12 h, and western blotting was performed. Cell culture supernatants were collected 48 h after treatment with LPS/ATP, and ELISA was performed to quantify the levels of (f) IL-1*β* and (g) IL-18. ^###^*p* < 0.001 vs. untreated cells; ^∗∗∗^*p* < 0.001 and ^∗^*p* < 0.05 vs. LPS/ATP-treated cells. UT: untreated cells.

We, next, examined whether the LPS/ATP-induced NLRP3 inflammasome was regulated by ER stress. Western blot analysis showed that GRP78, p-eIF2*α*, ATF4, and CHOP were remarkably increased at 9 h after LPS/ATP treatment, which was sustained until 18 h (Figure 3(a)). Total eIF2*α* was sustained for 24 h. However, PS decreased the expression of ER stress marker proteins in LPS/ATP-treated BV2 microglia cells in a concentration-dependent manner (Figure 3(b)), indicating that PS alleviates LPS/ATP-induced ER stress. In addition, the cells were stained for intracellular Ca^2+^ in the presence or absence of PS. As shown in Figure 3(c), intracellular Ca^2+^ accumulation was markedly upregulated by treatment with LPS/ATP at 12 h, which was remarkably downregulated in the presence of PS. A selective ER stress inhibitor, salubrinal, also resulted in the reduction of LPS/ATP-stimulated Ca^2+^ release (Figure 3(d)) and the downregulation of NLRP3 and ASC expression, concomitant with the restoration of pro-caspase-1 (Figure 3(e)). Furthermore, LPS/ATP-mediated IL-1*β* (from 632.7 ± 14.4 pg/mL to 341.9 ± 32.4 pg/mL, Figure 3(f)) and IL-18 (from 491.4 ± 46.2 pg/mL to 288.9 ± 36.8 pg/mL, Figure 3(g)) release was significantly downregulated by treatment with salubrinal, which indicates that ER stress-induced Ca^2+^ accumulation is a key regulator of NLRP3 inflammasome-mediated IL-1*β* and IL-18 release. Altogether, these results indicate that PS inhibit the LPS/ATP-induced NLRP3 inflammasome by attenuating ER stress-induced Ca^2+^ accumulation and subsequently reduced IL-1*β* and IL-18 release.

### 3.4. Excessive Ca^2+^ Accumulation Activates the NLRP3 Inflammasome through mtROS Production

**Figure 4:**
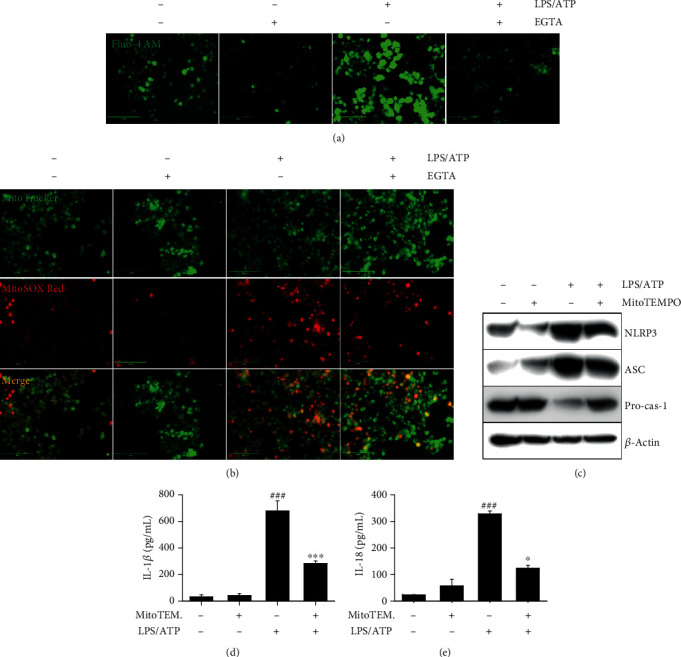
LPS/ATP-induced intracellular Ca^2+^ accumulation activates the NLRP3 inflammasome through mtROS production. BV2 microglia cells were pretreated with 2 mM EGTA for 2 h prior to stimulation with LPS/ATP for 12 h. The cells were stained with (a) 1 *μ*M Fluo-4 AM and (b) 0.5 *μ*M MitoTracker Green and 2 *μ*M MitoSOX Red. Cell images were captured by CELENA S Digital Imaging System. (c) The cells were pretreated with 50 *μ*M MitoTEMPO for 2 h prior to stimulation with LPS/ATP. Total protein was isolated at 12 h, and western blotting was performed. Cell culture supernatants were collected at 48 h, and ELISA was performed to quantify the levels of (d) IL-1*β* and (e) IL-18. ^∗∗∗^*p* < 0.001 and ^∗^*p* < 0.05 vs. untreated cells; ^###^*p* < 0.001 vs. LPS/ATP-treated cells.

To verify the relationship between Ca^2+^ and the NLPR3 inflammasome, we pretreated BV2 microglia cells with 2 mM EGTA, a Ca^2+^ chelator, for 2 h prior to stimulation with LPS/ATP for 12 h. As expected, LPS/ATP-induced Ca^2+^ levels were markedly decreased in the presence of EGTA (Figure 4(a)). In addition, LPS/ATP enhanced mtROS production; however, the production was also downregulated by treatment with EGTA (Figure 4(b)), indicating that intracellular Ca^2+^ accumulation promotes mtROS production in LPS/ATP-treated condition. We, next, elaborated the significance of Ca^2+^-induced mtROS production in the activation of the NLRP3 inflammasome. As shown in Figure 4(c), the mtROS scavenger, MitoTEMPO, strongly downregulated LPS/ATP-induced NLRP3 and ASC expression, accompanied by a remarkable increase in pro-caspase-1 expression. In addition, treatment with MitoTEMPO inhibited LPS/ATP-induced IL-1*β* (from 684.7 ± 76.7 pg/mL to 288.7 ± 19.7 pg/mL, Figure 4(d)) and IL-18 (from 331.0 ± 11.6 pg/mL to 126.2 ± 11.3 pg/mL, Figure 4(e)) release. Collectively, these results indicate that Ca^2+^-mediated mtROS plays a significant role in the NLRP3 inflammasome-induced IL-1*β* and IL-18 release.

### 3.5. PS Prevent Mitochondrial Membrane Depolarization and Thereby Inhibit mtROS Production

**Figure 5:**
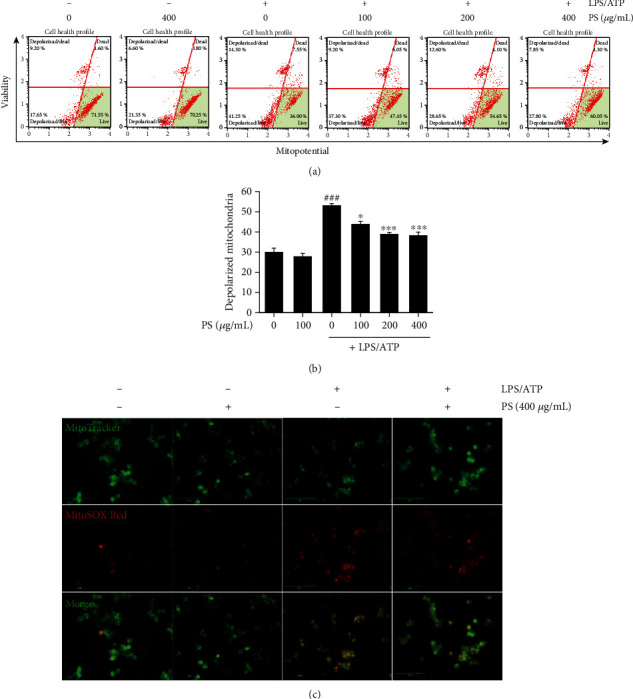
PS decrease mtROS production by stabilizing mitochondrial membrane integrity. (a) BV2 microglia cells were pretreated with the indicated concentrations of PS (0–400 *μ*g/mL) for 2 h prior to stimulation with LPS/ATP for 12 h. (a) Mitochondrial membrane depolarized cell populations were measured using a Muse MitoPotential Kit. (b) Total populations of mitochondrial membrane depolarized cells were represented. (c) In a parallel experiment, the cells were stained with 0.5 *μ*M MitoTracker Green and 2 *μ*M MitoSOX Red, and cell images were captured by CELENA S Digital Imaging System. ^###^*p* < 0.001 vs. untreated cells; ^∗∗∗^*p* < 0.001 and ^∗^*p* < 0.05 vs. LPS/ATP-treated cells.

As mitochondrial membrane depolarization is a key regulator of mtROS production, we investigated the effect of PS on mitochondria membrane depolarization and mtROS production in response to LPS/ATP using flow cytometry (Figure 5(a)). The data showed that LPS/ATP significantly induced mitochondrial membrane depolarization (53.6% ± 1.1%); however, PS decreased LPS/ATP-induced mitochondria membrane depolarization in a concentration-dependent manner (44.3% ± 1.5%, 39.3% ± 0.9%, and 38.7% ± 1.8% at 100, 200, and 400 *μ*g/mL, respectively) (Figure 5(b)). In addition, PS resulted in a remarkable decrease in mtROS production induced by LPS/ATP treatment (Figure 5(c)). Collectively, these data indicate that PS inhibit LPS/ATP-induced mitochondrial membrane depolarization by stabilizing membrane integrity, leading to the downregulation of mtROS production.

### 3.6. PS Inhibit Ca^2+^ Accumulation and Subsequent mtROS Production in the Early Stage, Not through ER Stress

**Figure 6:**
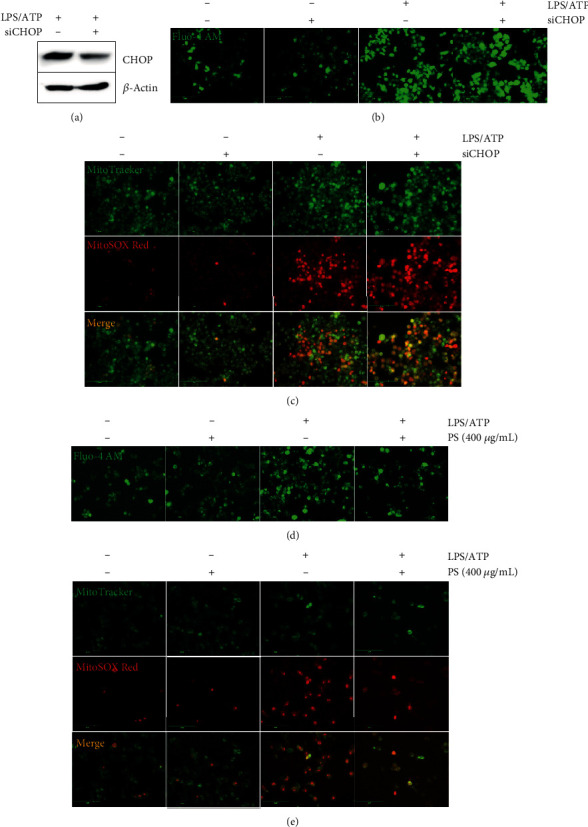
PS inhibit NLRP3 inflammasome in the early stage, independently from ER stress. BV2 microglia cells were transiently transfected with *CHOP* siRNA for 48 h and treated with LPS/ATP. (a) Transfection efficiency was shown by western blotting after LPS/ATP treatment. The cells were treated with LPS/ATP for 1 h and stained with (b) Fluo-4 AM and (c) 0.5 *μ*M MitoTracker Green and with 2 *μ*M MitoSOX Red. In a parallel experiment, BV2 microglia cells were pretreated with 400 *μ*g/mL PS for 2 h prior to stimulation with LPS/ATP for 1 h. The cells were stained with (d) 1 *μ*M Fluo-4 AM and (e) 0.5 *μ*M MitoTracker Green and 2 *μ*M MitoSOX Red. Cell images were captured using CELENA S Digital Imaging System.

To verify the possible involvement of ER stress in LPS/ATP-induced Ca^2+^ accumulation and mtROS production in the early stage, BV2 microglia cells were transfected with *CHOP* siRNA for 48 h and then treated with LPS/ATP for 1 h. Transfection of *CHOP* siRNA remarkably decreased CHOP expression (Figure 6(a)). Unexpectedly, the transient knockdown of *CHOP* did not downregulate excessive Ca^2+^ accumulation (Figure 6(b)) and mtROS production (Figure 6(c)) in the early stage, indicating that other signaling pathways stimulate LPS/ATP-induced Ca^2+^ accumulation and mtROS production in the early stage. These results indicated that ER stress may not regulate the NLRP3 inflammasome in the early stage. However, PS significantly inhibited LPS/ATP-induced Ca^2+^ accumulation (Figure 6(d)) and mtROS production (Figure 6(e)) in the early stage. Collectively, these results indicate that PS inhibit LPS/ATP-mediated Ca^2+^ accumulation and mtROS production independently from the ER stress in the early stage, which may play a role in downregulation of the NLRP3 inflammasome.

### 3.7. PS Inhibit LPS/ATP-Induced Nuclear Translocation of NF-*κ*B in the Early Stage

**Figure 7:**
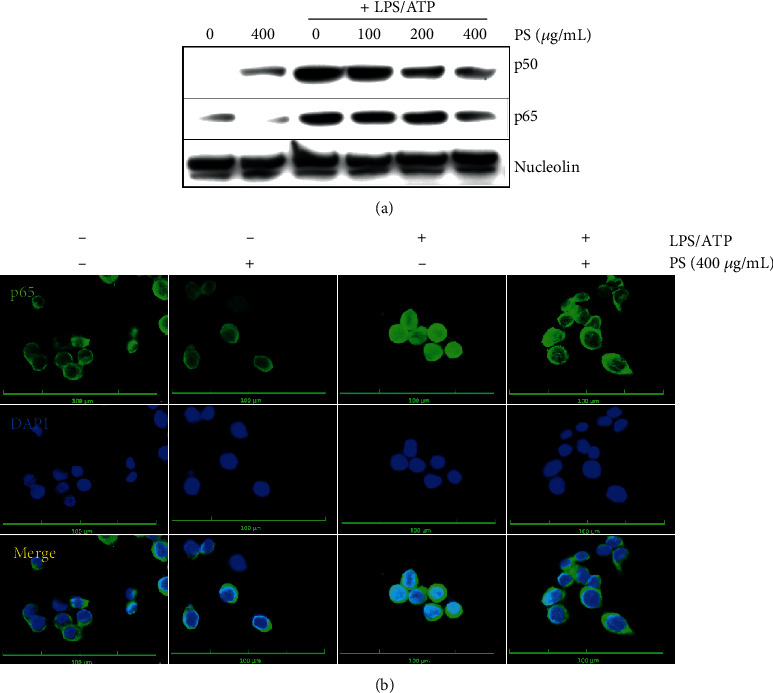
PS inhibit nuclear translocation of NF-*κ*B. BV2 microglia cells were pretreated with the indicated concentrations of PS for 2 h prior to LPS/ATP. (a) Nuclear proteins were extracted at 1 h, and western blotting was performed. (b) In a parallel experiment, the cells were immunostained using NF-*κ*B p65 antibody conjugated with Alexa Fluor 488. Cell images were captured by CELENA S Digital Imaging System.

To address whether PS attenuate LPS/ATP-induced NF-*κ*B and subsequently downregulate the NLRP3 inflammasome, BV2 microglia cells were pretreated with the indicated concentrations of PS before treatment with LPS/ATP, and the expression of NF-*κ*B p50 and p65 was investigated. As shown in Figure 7(a), PS markedly downregulated LPS/ATP-induced nuclear translocation of NF-*κ*B p50 and p65 in a concentration-dependent manner. Immunofluorescence data also confirmed that LPS/ATP remarkably enhanced the nuclear translocation of NF-*κ*B p65; however, PS downregulated its nuclear translocation (Figure 7(b)).

### 3.8. PS Downregulate LPS/ATP-Induced NLRP3 Inflammasome in the Early Stage through Inhibition of the NF-*κ*B Signaling Pathway, Leading to the Reduction of IL-1*β* and IL-18 Release

**Figure 8:**
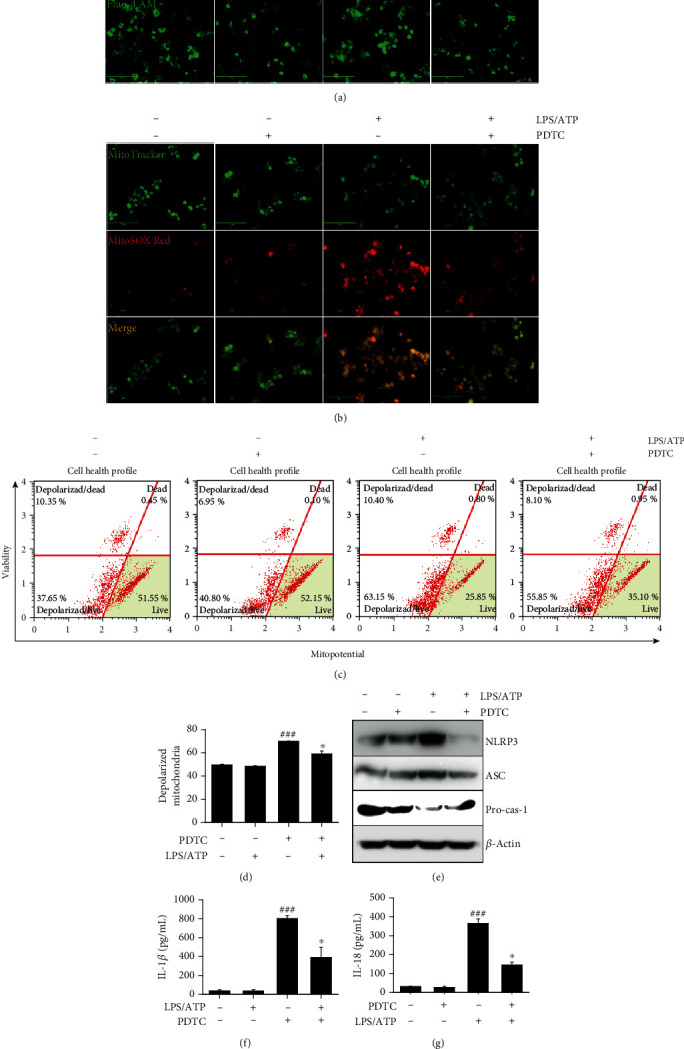
The NF-*κ*B signaling pathway activates LPS/ATP-induced NLRP3 formation and subsequent IL-1*β* and IL-18 release in the early stage. (a) BV2 microglia cells were pretreated with 10 *μ*M PDTC for 2 h prior to stimulation with LPS/ATP for 1 h. The cells were stained with (a) 1 *μ*M Fluo-4 AM and (b) 0.5 *μ*M MitoTracker Green and 2 *μ*M MitoSOX Red. Cell images were captured by CELENA S Digital Imaging System. (c) In a parallel experiment, mitochondrial membrane depolarization was measured using a Muse MitoPotential Kit. (d) The total populations of mitochondrial membrane depolarized cells were shown. (e) Total proteins were isolated at 12 h, and western blotting was performed. Cell culture supernatant was collected at 48 h, and ELISA was performed to quantify the levels of (e) IL-1*β* and (f) IL-18. ^###^*p* < 0.001 vs. untreated cells; ^∗^*p* < 0.05 vs. LPS/ATP-treated cells. PDTC: pyrrolidine dithiocarbamate.

As we elucidated that PS inhibited the nuclear translocation of NF-*κ*B, we confirmed whether the NF-*κ*B signaling pathway activated LPS/ATP-induced NLRP3 formation in the early stage by increasing intracellular Ca^2+^ accumulation and mtROS generation and consequently promoted the release of IL-1*β* and IL-18. Pretreatment with an NF-*κ*B inhibitor PDTC dramatically decreased LPS/ATP-induced Ca^2+^ accumulation (Figure 8(a)) and mtROS production (Figure 8(b)). This was further confirmed by assessing mitochondrial membrane depolarization using flow cytometry (Figure 8(c)). LPS/ATP-induced mitochondrial membrane depolarization (66.9% ± 0.3%) was significantly downregulated in the presence of PDTC (58.9% ± 2.6%) (Figure 8(d)), indicating that NF-*κ*B stimulates LPS/ATP-induced mitochondrial membrane depolarization in the early stage. Additionally, treatment with PDTC downregulated the expression of NLRP3 and ASC, concomitant with the restoration of pro-caspase-1 (Figure 8(e)). We also found that the inhibition of NF-*κ*B by PDTC downregulated LPS/ATP-induced IL-1*β* (from 800.8 ± 33.4 pg/mL to 389.5 ± 107.2 pg/mL, Figure 8(f)) and IL-18 (from 365.5 ± 23.4 pg/mL to 144.2 ± 15.7 pg/mL, Figure 8(g)) release. Collectively, these data indicate that the NF-*κ*B signaling pathway upregulates NLRP3 inflammasome-induced IL-1*β* and IL-18 release in the early stage by increasing Ca^2+^ accumulation and mtROS production.

## 4. Discussion

**Figure 9:**
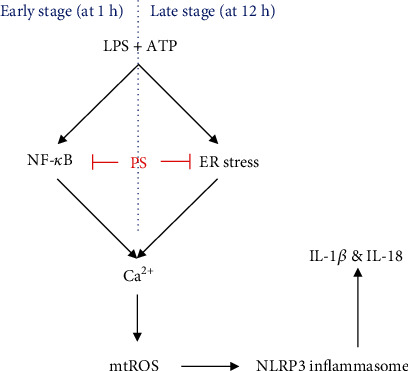
PS inhibit the NLRP3 inflammasome through two different pathways. To activate the NLRP3 inflammasome, NF-*κ*B was stimulated in the early stage (1 h after treatment with LPS/ATP) and ER stress was stimulated in the late stage (12 h after treatment with LPS/ATP); both pathways enhanced Ca^2+^ accumulation and the subsequent mtROS generation, which lead to IL-1*β* and IL-18 release. PS inhibited the NF-*κ*B signaling pathway in the early stage and downregulated ER stress in the late stage, which reduced intracellular Ca^2+^-mediated mtROS production and subsequently inhibited NLRP3 inflammasome-induced IL-1*β* and IL-18 release. NLRP3: NOD, LRR, and pyrin domain-containing protein 3; NF-*κ*B: nuclear factor-*κ*B; ER: endoplasmic reticulum; mtROS: mitochondrial reactive oxygen species.

The NLRP3 inflammasome is associated with several human disorders, including Alzheimer's disease, atherosclerosis, and allergy airway inflammation [3]. PS has been used as a medicinal food source; however, most of the pharmaceutical properties have not been confirmed. Our recent studies confirmed that PS inhibited melanogenesis in B16F10 melanoma cells and zebrafish larvae [25], H_2_O_2_-induced oxidative stress in HaCaT keratinocytes [24], and LPS-induced endotoxic shock [23]; thus, PS can be considered as a potent pharmacological candidate for a broad spectrum of diseases. In this study, we examined the anti-inflammatory properties of PS in the LPS/ATP-induced NLRP3 inflammasome. We demonstrated that PS inhibited NLRP3 inflammasome-related cell death in BV2 microglia cells by downregulating Ca^2+^ accumulation and mtROS generation. In particular, stimulation with LPS/ATP primed the NLRP3 inflammasome at two different time points: through the NF-*κ*B pathway in the early stage (at 1 h) and through the ER stress-mediated pathway in the late stage (at 12 h). Both pathways were associated with the Ca^2+^ accumulation and mtROS generation, which was significantly inhibited in the presence of PS (Figure 9).

The NLRP3 inflammasome is a critical nexus by promoting the maturation and/or activation of inflammatory IL-1*β* and IL-18 against pathogens and a diverse range of innate immune stimuli [1]. Activation of the NLRP3 inflammasome leads to the complex assembly through the conformational changes of the relevant proteins, leading to the cleavage of pro-caspase-1 [26]. ATP is a general requirement for NLRP3 inflammasome activation and assembly through intracellular Ca^2+^ mobilization [27, 28]. In this study, we also found that cytosolic Ca^2+^ levels were significantly upregulated in response to LPS/ATP in both the early and late stages and subsequently activated the NLRP3 inflammation-induced IL-1*β* and IL-18 release, which was remarkably inhibited by PS. Katsnelson et al. [29] demonstrated that increased cytosolic Ca^2+^ levels were neither necessary nor sufficient for the formation of the NLRP3 inflammasome cascade during activation by endogenous ATP-gated P2X7 receptor channel. However, in this study, the chelation of cytosolic Ca^2+^ using EGTA resulted in a significant impairment of mtROS production, which resulted in the inhibition of the NLRP3 inflammasome, indicating that Ca^2+^ accumulation stimulated the NLRP3 inflammasome by increasing mtROS production. Xu and colleagues [20, 30] also showed that the accumulated Ca^2+^ was transported to the mitochondrial matrix and resulted in mitochondrial dysfunction and excessive mtROS production, which subsequently promoted activation of the NLRP3 inflammasome. Recently, administration of 20 mg/kg MitoTEMPO prior to exposure to ozone (2.5 ppm for 3 h) resulted in reduced levels of total malondialdehyde in bronchoalveolar lavage fluid in C57/BL6 mice through inhibition of the NLRP3 inflammasome [30]. Consistent with previous data, we found that pretreatment with MitoTEMPO significantly lowered the activation of the NLRP3 inflammasome, followed by IL-1*β* and IL-18 release, which indicated the significance of Ca^2+^ mobilization and mtROS production in the NLRP3 inflammasome-induced IL-1*β* and IL-18 release. In this study, we found that PS inhibited LPS/ATP-induced cell death, with concomitant restraint of the NLRP3 inflammasome, owing to a significant decrease in Ca^2+^ accumulation and mtROS production.

ER stress-mediated UPR is also associated with activation of the NLRP3 inflammasome and the subsequent release of IL-1*β* [15]. Lebeaupin and colleagues [31] demonstrated that the ER stress signaling pathway increased NLRP3 inflammasome activation and hepatocyte death and consequently led to nonalcoholic fatty liver disease. In addition, LPS activated IRE1*α* and PERK, including the overexpression of CHOP, which upregulated NLRP3-medicated caspase-1 activation [15]. The above data showed that ER stress was a key regulator of the activation of the NLRP3 inflammasome. In this study, we confirmed that treatment with LPS/ATP upregulated the expression of ER stress marker proteins at 12 h accompanied by high levels of Ca^2+^ accumulation and mtROS production, which enhanced NLRP3 inflammation-induced IL-1*β* and IL-18 release. In addition, an ER stress inhibitor, salubrinal, inhibited the LPS/ATP-induced NLRP3 inflammasome, indicating that ER stress plays a crucial role in the activation of the NLRP3 inflammasome. In this study, PS strongly inhibited LPS/ATP-induced ER stress, resulting in downregulation of the NLRP3 inflammasome; meanwhile, Ca^2+^ accumulation and mtROS dramatically declined. Nevertheless, transient knockdown of *CHOP* showed that Ca^2+^ accumulation and mtROS production in the early stage (1 h after treatment with LPS/ATP) were independent of ER stress, which indicated that other signaling pathways were related to regulation of the NLRP3 inflammasome before activation of the ER stress response. Therefore, further studies are needed to evaluate which signaling pathways regulate Ca^2+^ accumulation and mtROS-induced NLRP3 activation in the early stage. Recent studies showed that human monocytes released mature IL-1*β* and IL-18 in response to LPS through the activation of the NLRP3 inflammasome. LPS promotes endogenous ATP production, which in turn activates the P2X7 receptor, resulting in NLRP3 inflammasome-induced maturation and releases of IL-1*β* and IL-18 [32, 33]. This alternative pathway is not required for K^+^ efflux, but Ca^2+^ mobilization-mediated mtROS production is essential for NLRP3 inflammation activation [34, 35]. In this study, we found that a specific NF-*κ*B inhibitor, PDTC, inhibited the NLRP3 inflammasome and, subsequently, IL-1*β* and IL-18 maturation through Ca^2+^ accumulation and mtROS production, before the activation of ER stress response.

## 5. Conclusions

In conclusion, this study revealed that PS inhibited the LPS/ATP-induced NLRP3 inflammasome by inhibiting the NF-*κ*B signaling pathway in the early stage and the ER stress response in the late stage, accompanied with downregulation of Ca^2+^ accumulation and mtROS production. Finally, PS attenuated the LPS/ATP-induced IL-1*β* and IL-18 release, indicating that PS may be a potent anti-inflammatory candidate targeting the NLRP3 inflammasome. However, the mechanism through which NF-*κ*B regulates Ca^2+^ accumulation and mtROS production in the early stage is still undiscovered. In addition, Lentschat et al. [36] demonstrated that LPS can be internalized into macrophages within 1 h and stimulate caspase-11-mediated noncanonical NLRP3 pathway. Therefore, it will be confirmed whether PS regulates the noncanonical NLRP3 inflammasome pathways. Finally, as dietary anthocyanins prevent many inflammatory diseases in vivo, such as ulcerative colitis and cerebral ischemia-reperfusion injury [37, 38], it should be determined whether PS is effective in vivo against NLRP3 inflammasome-mediated inflammation.

## Figures and Tables

**Table 1:** Primers and PCR conditions used in this experiment.

Gene^∗^	Primer sequence (5′-3′)	Amplicon size	*T*_m_	Cycle no.
*NLRP3*	F 5′-ATTACCCGCCCGAGAAAGG-3′ R 5′-TCGCAGCAAAGATCCACACAG-3′	120 bp	58°C	27
*ASC*	F 5′-GCAACTGCGAGAAGGCTAT-3′ R 5′-CTGGTCCACAAAGTGTCCTG-3′	236 bp	58°C	27
*IL-1β*	F 5′-GCCCATCCTCTGTGACTCAT-3′ R 5′-AGGCCACAGGTATTTTGTCG-3′	210 bp	65°C	27
*IL-18*	F 5′-AGACAGCCTGTGTTCGAG-3′ R 5′-AAGGAGGGTCACAGCCAGTC-3′	100 bp	63°C	27
*GAPDH*	F 5′-CACCACCCTGTTGCTGTAGC-3′ R 5′-ACCACAGTCCATGCCATCAC-3′	123 bp	63°C	23

bp: base pair; *T*_m_: melting temperature. ^∗^NLRP3: NATCH, LRR, and PYD domain-containing protein 3; ASC: apoptosis-associated speck-like protein containing a CARD; IL-1*β*: interleukin 1 beta; IL-18: interleukin-18; GAPDH: glyceraldehyde 3-phosphate dehydrogenase.

## Data Availability

The data that support the findings of this study are available from the corresponding author upon reasonable request.

## Abbreviations

ASC: Apoptosis-associated speck-like protein containing a CARD
ATP: Adenosine 5′-triphosphate disodium salt hydrate
ATF6: Activating transcription factor 6
BiP: Immunoglobulin protein
CHO: CCAAT-enhancer-binding protein (C/EBP) homologous protein
DMEM: Dulbecco's modified Eagle's medium
eIF2*α*: Eukaryotic translation initiation factor 2*α*
ELISA: Enzyme-linked immunosorbent assay
ER: Endoplasmic reticulum
ERAD: Endoplasmic reticulum-associated protein degradation
FBS: Fetal bovine serum
GAPDH: Glyceraldehyde 3-phosphate dehydrogenase
IL: Interleukin
IRE1: Inositol-requiring kinase 1
LPS: Lipopolysaccharide
LPS/ATP: Lipopolysaccharide and adenosine 5′-triphosphate disodium salt hydrate
mtROS: Mitochondrial reactive oxygen species
MTT: 3-(4,5-Dimethylthiazol-2-yl)-2,5-diphenyl-tetrazolium bromide
NLRP3: NATCH, LRR, and PYD domain-containing protein 3
PBS: Phosphate-buffered saline
PERK: PKR-like endoplasmic reticulum kinase
PDTC: Pyrrolidine dithiocarbamate
PKR: Double-stranded RNA-activated protein kinase
PS: Anthocyanins from the petals of *Hibiscus syriacus* L.
RT-PCR: Reverse transcriptase polymerase chain reaction
siRNA: Small interfering RNA
UPR: Unfolded protein response.

## Supplementary Materials

Supplementary Materials Supplementary Figure S1: triplicate data (A) and densitometric analysis (B) of Figure 2(a). Supplementary Figure S2: triplicate data (A) and densitometric analysis (B) of Figure 2(b). Supplementary Figure S3: triplicate data (A) and densitometric analysis (B) of Figure 2(c). Supplementary Figure S4: triplicate data (A) and densitometric analysis (B) of Figure 2(d). Supplementary Figure S5: triplicate data (A) and densitometric analysis (B) of Figure 3(a). Supplementary Figure S6: triplicate data (A) and densitometric analysis (B) of Figure 3(b). Supplementary Figure S7: triplicate data (A) and densitometric analysis (B) of Figure 3(e). Supplementary Figure S8: triplicate data (A) and densitometric analysis (B) of Figure 4(c). Supplementary Figure S9: triplicate data (A) and densitometric analysis (B) of Figure 7(a). Supplementary Figure S10: triplicate data (A) and densitometric analysis (B) of Figure 8(d).

## References

[1] Latz E., Xiao T. S., Stutz A. (2013). Activation and regulation of the inflammasomes. *Nature Reviews Immunology*.

[2] Jo E.-K., Kim J. K., Shin D. M., Sasakawa C. (2016). Molecular mechanisms regulating NLRP3 inflammasome activation. *Cellular & Molecular Immunology*.

[3] Mangan M. S. J., Olhava E. J., Roush W. R., Seidel H. M., Glick G. D., Latz E. (2018). Targeting the NLRP3 inflammasome in inflammatory diseases. *Nature Reviews Drug Discovery*.

[4] Wang Z., Hu W., Lu C. (2018). Targeting NLRP3 (nucleotide-binding domain, leucine-rich–containing family, pyrin domain–containing-3) inflammasome in cardiovascular disorders. *Arteriosclerosis, Thrombosis, and Vascular Biology*.

[5] Kelley N., Jeltema D., Duan Y., He Y. (2019). The NLRP3 inflammasome: an overview of mechanisms of activation and regulation. *International Journal of Molecular Sciences*.

[6] Malik A., Kanneganti T.-D. (2017). Inflammasome activation and assembly at a glance. *Journal of Cell Science*.

[7] Franchi L., Eigenbrod T., Muñoz-Planillo R., Nuñez G. (2009). The inflammasome: a caspase-1-activation platform that regulates immune responses and disease pathogenesis. *Nature Immunology*.

[8] Wang M., Kaufman R. J. (2016). Protein misfolding in the endoplasmic reticulum as a conduit to human disease. *Nature*.

[9] Hetz C., Papa F. R. (2018). The unfolded protein response and cell fate control. *Molecular Cell*.

[10] Rutkowski D. T., Kaufman R. J. (2004). A trip to the ER: coping with stress. *Trends in Cell Biology*.

[11] Lee A. S. (2005). The ER chaperone and signaling regulator GRP78/BiP as a monitor of endoplasmic reticulum stress. *Methods*.

[12] Schönthal A. H. (2012). Endoplasmic reticulum stress: its role in disease and novel prospects for therapy. *Scientifica*.

[13] Kaira K., Toyoda M., Shimizu A. (2016). Expression of ER stress markers (GRP78/BiP and PERK) in patients with tongue cancer. *Neoplasma*.

[14] Casas C. (2017). GRP78 at the centre of the stage in cancer and neuroprotection. *Frontiers in Neuroscience*.

[15] Menu P., Mayor A., Zhou R. (2012). ER stress activates the NLRP3 inflammasome via an UPR-independent pathway. *Cell Death & Disease*.

[16] Adams C. J., Kopp M. C., Larburu N., Nowak P. R., Ali M. M. U. (2019). Structure and molecular mechanism of ER stress signaling by the unfolded protein response signal activator IRE1. *Frontiers in Molecular Biosciences*.

[17] Hamilton C., Anand P. K. (2019). Right place, right time: localisation and assembly of the NLRP3 inflammasome. *F1000Research*.

[18] Han C. Y., Rho H. S., Kim A. (2018). FXR inhibits endoplasmic reticulum stress-induced NLRP3 inflammasome in hepatocytes and ameliorates liver injury. *Cell Reports*.

[19] Yang Y., Wang H., Kouadir M., Song H., Shi F. (2019). Recent advances in the mechanisms of NLRP3 inflammasome activation and its inhibitors. *Cell Death & Disease*.

[20] Xu Z., Zhang D., He X., Huang Y., Shao H. (2016). Transport of calcium ions into mitochondria. *Current Genomics*.

[21] Feno S., Butera G., Reane D. V., Rizzuto R., Raffaello A. (2019). Crosstalk between calcium and ROS in pathophysiological conditions. *Oxidative Medicine and Cellular Longevity*.

[22] Kwon H.-Y., Kim J.-H., Kim S.-H., Park J.-M., Lee H. (2016). The complete chloroplast genome sequence of Hibiscus syriacus. *Mitochondrial DNA A DNA Mapping, Sequencing, and Analysis*.

[23] MadurangaKarunarathne W. A. H., TaeLee K., HyunChoi Y., Cheng-YunJin G.-Y. K. (2020). Anthocyanins isolated from _Hibiscus syriacus_ L. attenuate lipopolysaccharide-induced inflammation and endotoxic shock by inhibiting the TLR4/MD2-mediated NF-*κ*B signaling pathway. *Phytomedicine*.

[24] Molagoda I. M. N., Lee K. T., Choi Y. H., Kim G.-Y. (2020). Anthocyanins from Hibiscus syriacus L. inhibit oxidative stress-mediated apoptosis by activating the Nrf2/HO-1 signaling pathway. *Antioxidants (Basel, Switzerland)*.

[25] Karunarathne W. A. H. M., Molagoda I. M. N., Park S. R. (2019). Anthocyanins from Hibiscus syriacus L. inhibit melanogenesis by activating the ERK signaling pathway. *Biomolecules*.

[26] Elliott E. I., Sutterwala F. S. (2015). Initiation and perpetuation of NLRP3 inflammasome activation and assembly. *Immunological Reviews*.

[27] Murakami T., Ockinger J., Yu J. (2012). Critical role for calcium mobilization in activation of the NLRP3 inflammasome. *Proceedings of the National Academy of Sciences of the United States of America*.

[28] Muñoz-Planillo R., Kuffa P., Martínez-Colón G., Smith B. L., Rajendiran T. M., Núñez G. (2013). K^+^ efflux is the common trigger of NLRP3 inflammasome activation by bacterial toxins and particulate matter. *Immunity*.

[29] Katsnelson M. A., Rucker L. G., Russo H. M., Dubyak G. R. (2015). K^+^ efflux agonists induce NLRP3 inflammasome activation independently of Ca^2+^ signaling. *Journal of Immunology*.

[30] Xu M., Wang L., Wang M. (2019). Mitochondrial ROS and NLRP3 inflammasome in acute ozone-induced murine model of airway inflammation and bronchial hyperresponsiveness. *Free Radicical Research*.

[31] Lebeaupin C., Proics E., de Bieville C. H. D. (2015). ER stress induces NLRP3 inflammasome activation and hepatocyte death. *Cell Death & Disease*.

[32] Piccini A., Carta S., Tassi S., Lasiglié D., Fossati G., Rubartelli A. (2008). ATP is released by monocytes stimulated with pathogen-sensing receptor ligands and induces IL-1beta and IL-18 secretion in an autocrine way. *Proc Natlional Academy of Sciences of the United States of America*.

[33] He Y., Franchi L., Nunez G. (2012). TLR agonists stimulate Nlrp3-dependent IL-1beta production independently of the purinergic P2X7 receptor in dendritic cells and in vivo. *Journal of Immunology*.

[34] Gaidt M. M., Ebert T. S., Chauhan D. (2016). Human monocytes engage an alternative inflammasome pathway. *Immunity*.

[35] Horng T. (2014). Calcium signaling and mitochondrial destabilization in the triggering of the NLRP3 inflammasome. *Trends in Immunology*.

[36] Lentschat A., el-Samalouti V. T., Schletter J. (1999). The internalization time course of a given lipopolysaccharide chemotype does not correspond to its activation kinetics in monocytes. *Infection and Immunity*.

[37] Cui H.-X., Chen J.-H., Li J.-W., Cheng F.-R., Yuan K. (2018). Protection of anthocyanin from Myrica rubra against cerebral ischemia-reperfusion injury via modulation of the TLR4/NF-*κ*B and NLRP3 pathways. *Molecules*.

[38] Li S., Wu B., Fu W., Reddivari L. (2019). The anti-inflammatory effects of dietary anthocyanins against ulcerative colitis. *International Journal of Molecular Sciences*.

