# Supplementary material for: Anthocyanins from Hibiscus syriacus L. Inhibit NLRP3 Inflammasome in BV2 Microglia Cells by Alleviating NF-κB- and ER Stress-Induced Ca2+ Accumulation and Mitochondrial ROS Production
Source: Oxid Med Cell Longev. 2021 Feb 4;2021:1246491. doi: 10.1155/2021/1246491 (PMC7878077; doi:10.1155/2021/1246491)
Supplement: Supplementary Materials — Supplementary Figure S1: triplicate data (A) and densitometric analysis (B) of Figure 2(a). Supplementary Figure S2: triplicate data (A) and densitometric analysis (B) of Figure 2(b). Supplementary Figure S3: triplicate data (A) and densitometric analysis (B) of Figure 2(c). Supplementary Figure S4: triplicate data (A) and densitometric analysis (B) of Figure 2(d). Supplementary Figure S5: triplicate data (A) and densitometric analysis (B) of Figure 3(a). Supplementary Figure S6: triplicate data (A) and densitometric analysis (B) of Figure 3(b). Supplementary Figure S7: triplicate data (A) and densitometric analysis (B) of Figure 3(e). Supplementary Figure S8: triplicate data (A) and densitometric analysis (B) of Figure 4(c). Supplementary Figure S9: triplicate data (A) and densitometric analysis (B) of Figure 7(a). Supplementary Figure S10: triplicate data (A) and densitometric analysis (B) of Figure 8(d). [file 1246491.f1.pdf]

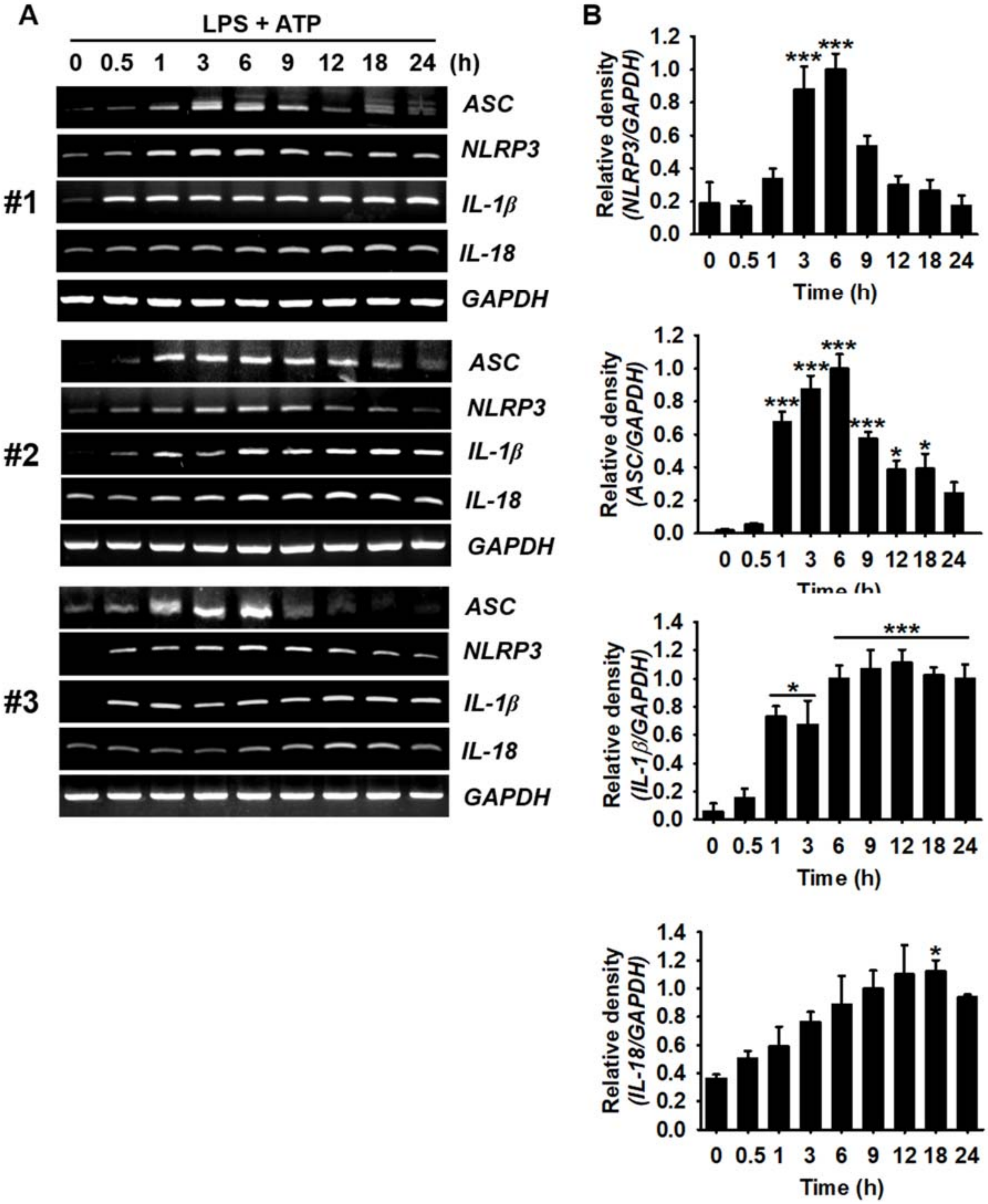

Supplementary Figure S1: Triplicate data (A) and densitometric analysis (B) of Figure 2A.

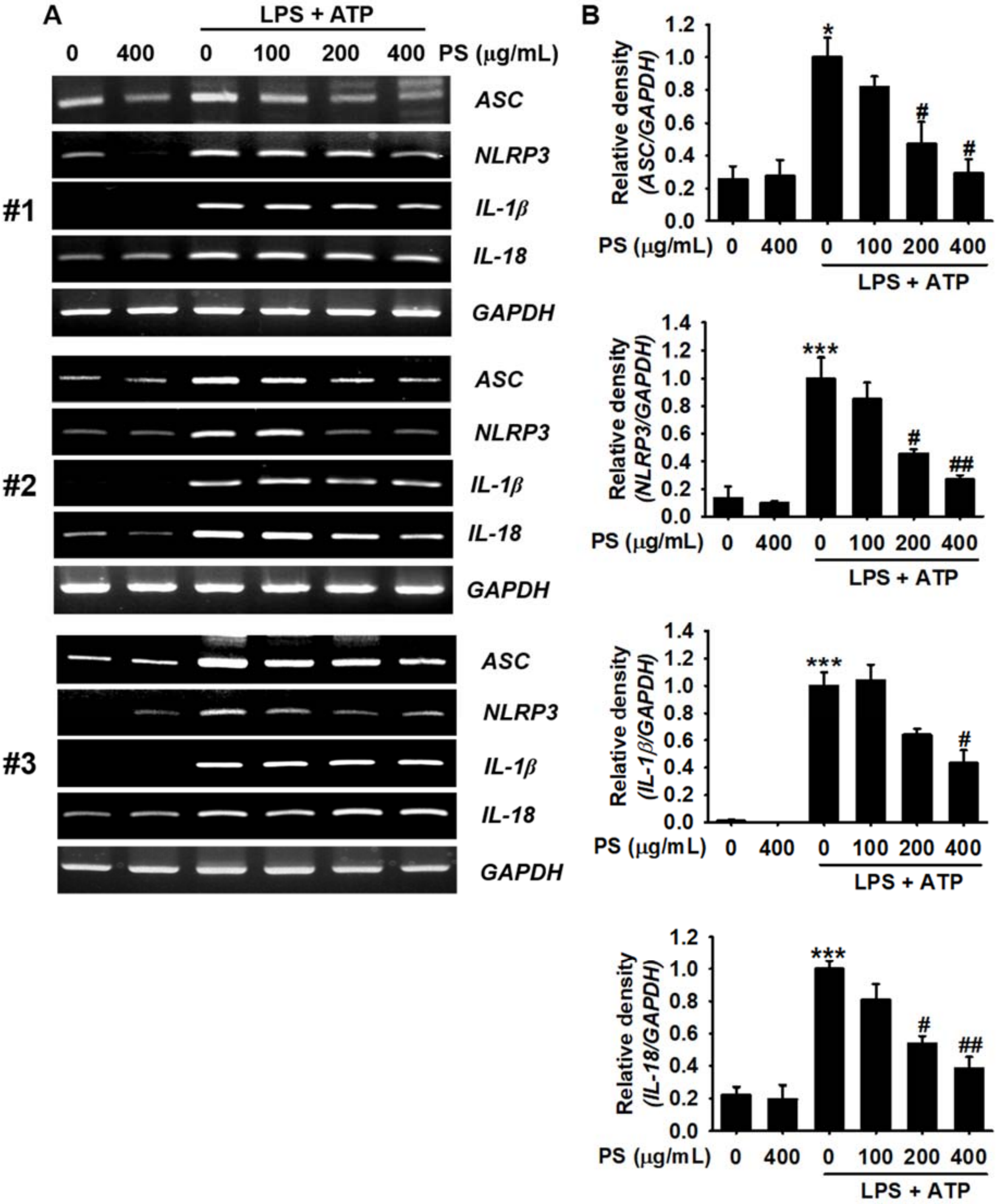

Supplementary Figure S2: Triplicate data (A) and densitometric analysis (B) of Figure 2B.

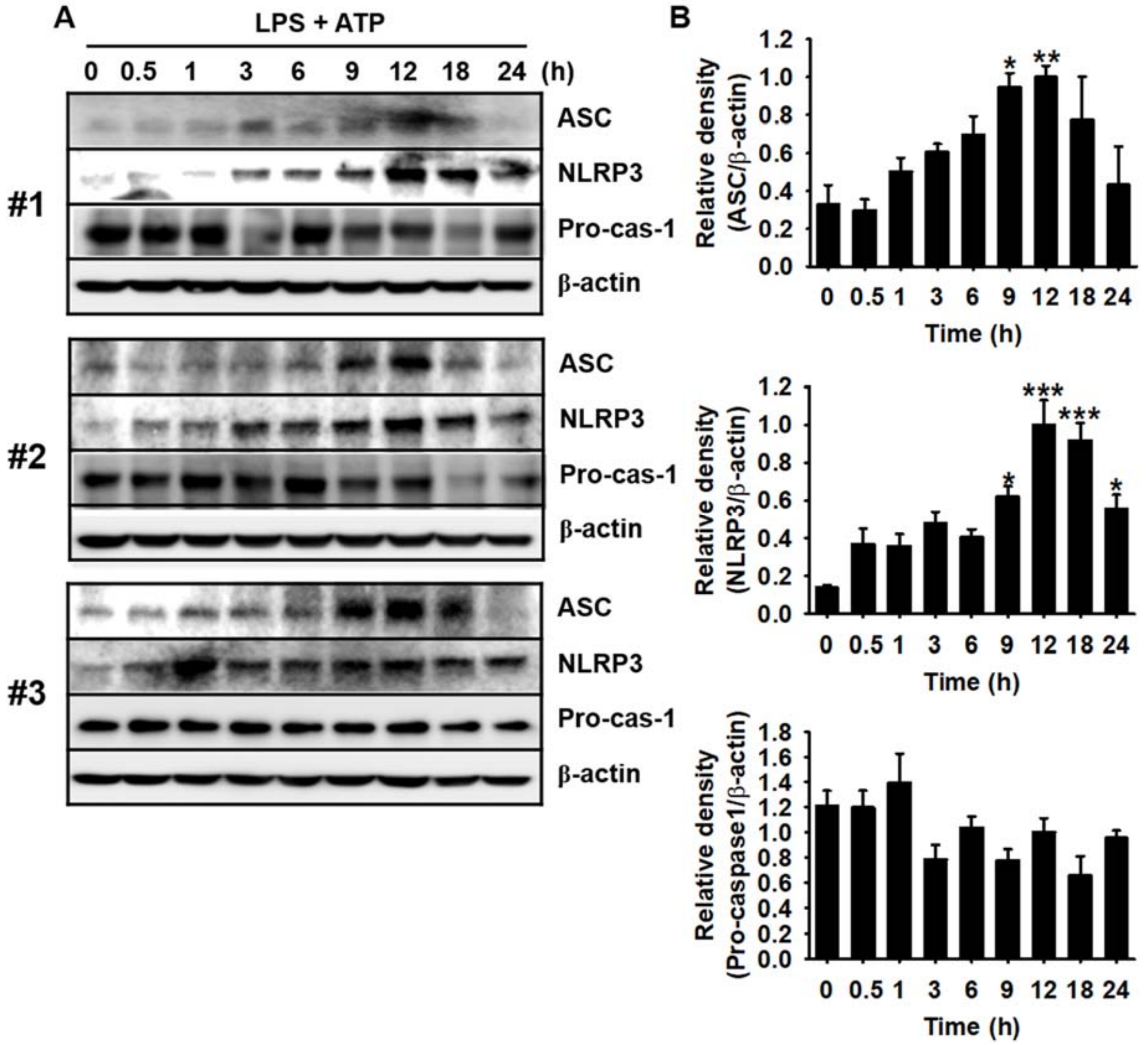

Supplementary Figure S3: Triplicate data (A) and densitometric analysis (B) of Figure 2C.

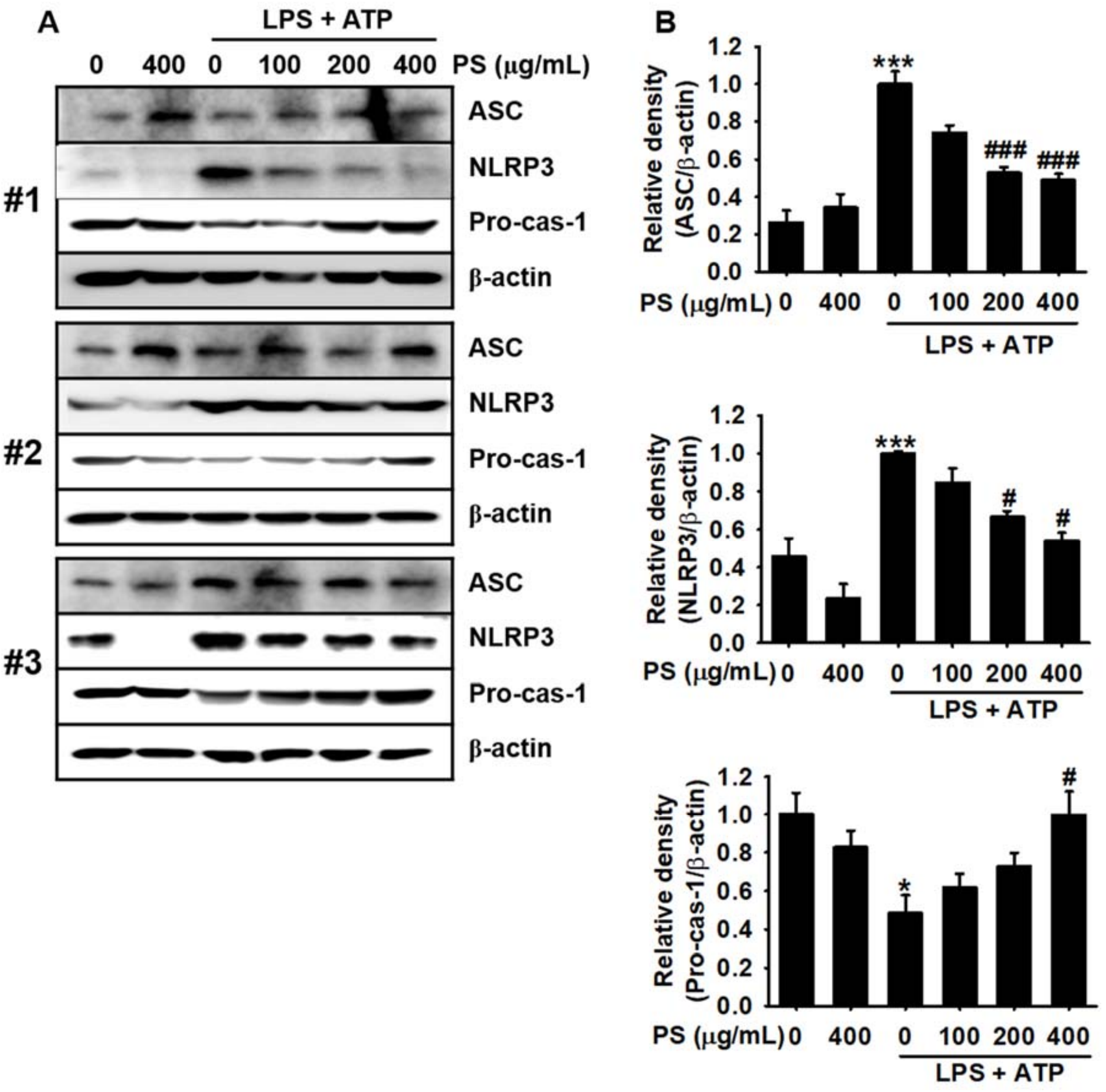

Supplementary Figure S4: Triplicate data (A) and densitometric analysis (B) of Figure 2D.

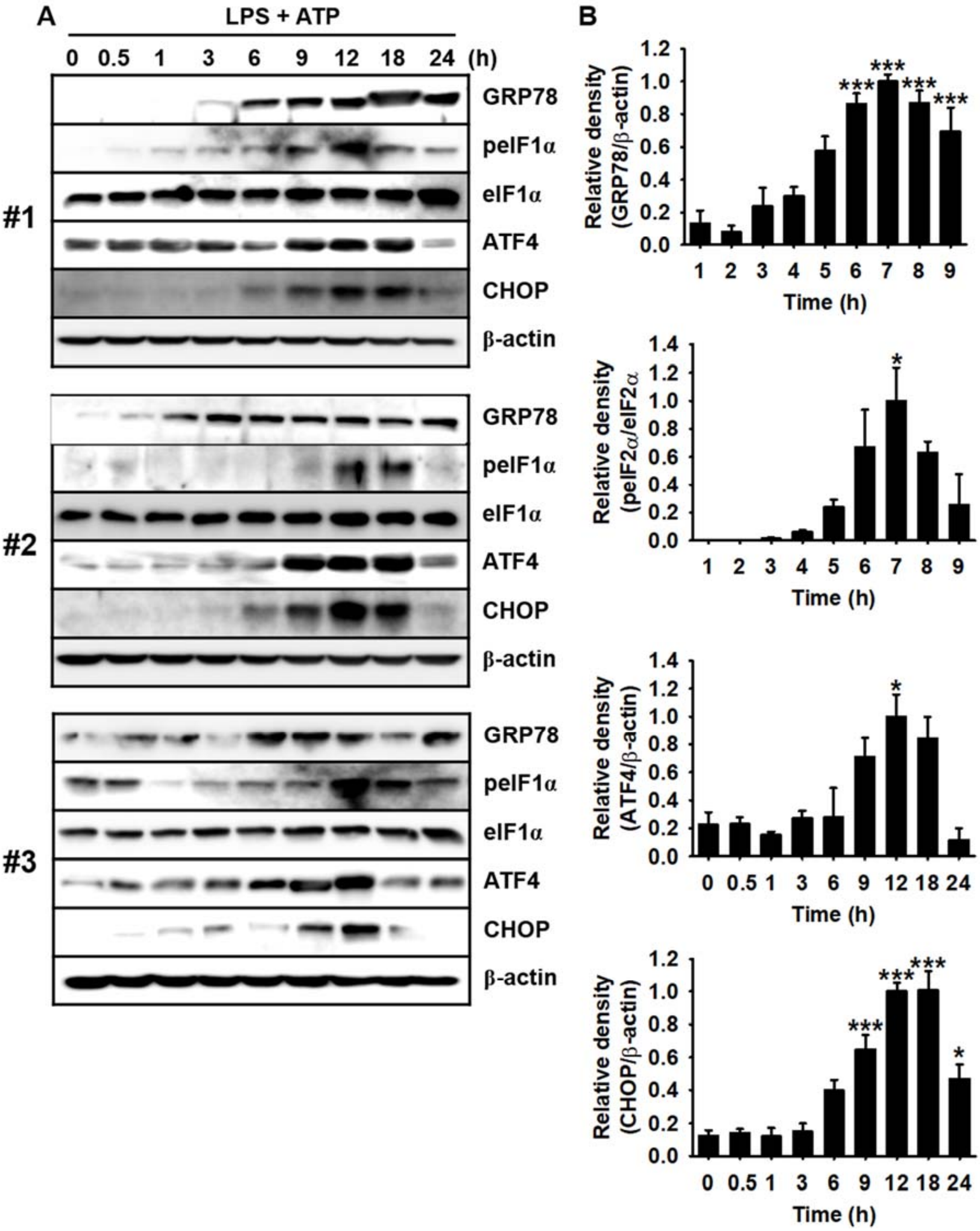

Supplementary Figure S5: Triplicate data (A) and densitometric analysis (B) of Figure 3A.

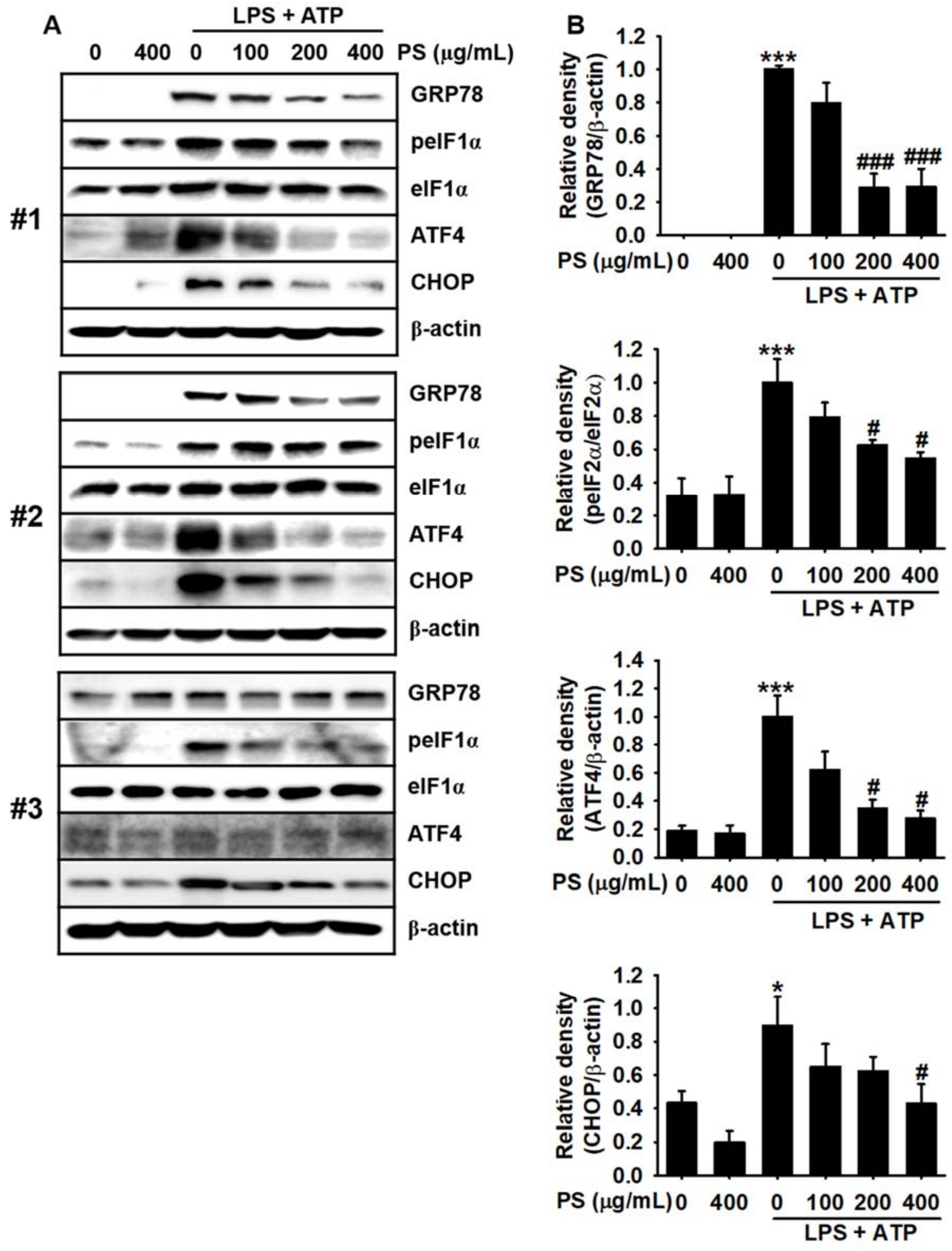

Supplementary Figure S6: Triplicate data (A) and densitometric analysis (B) of Figure 3B.

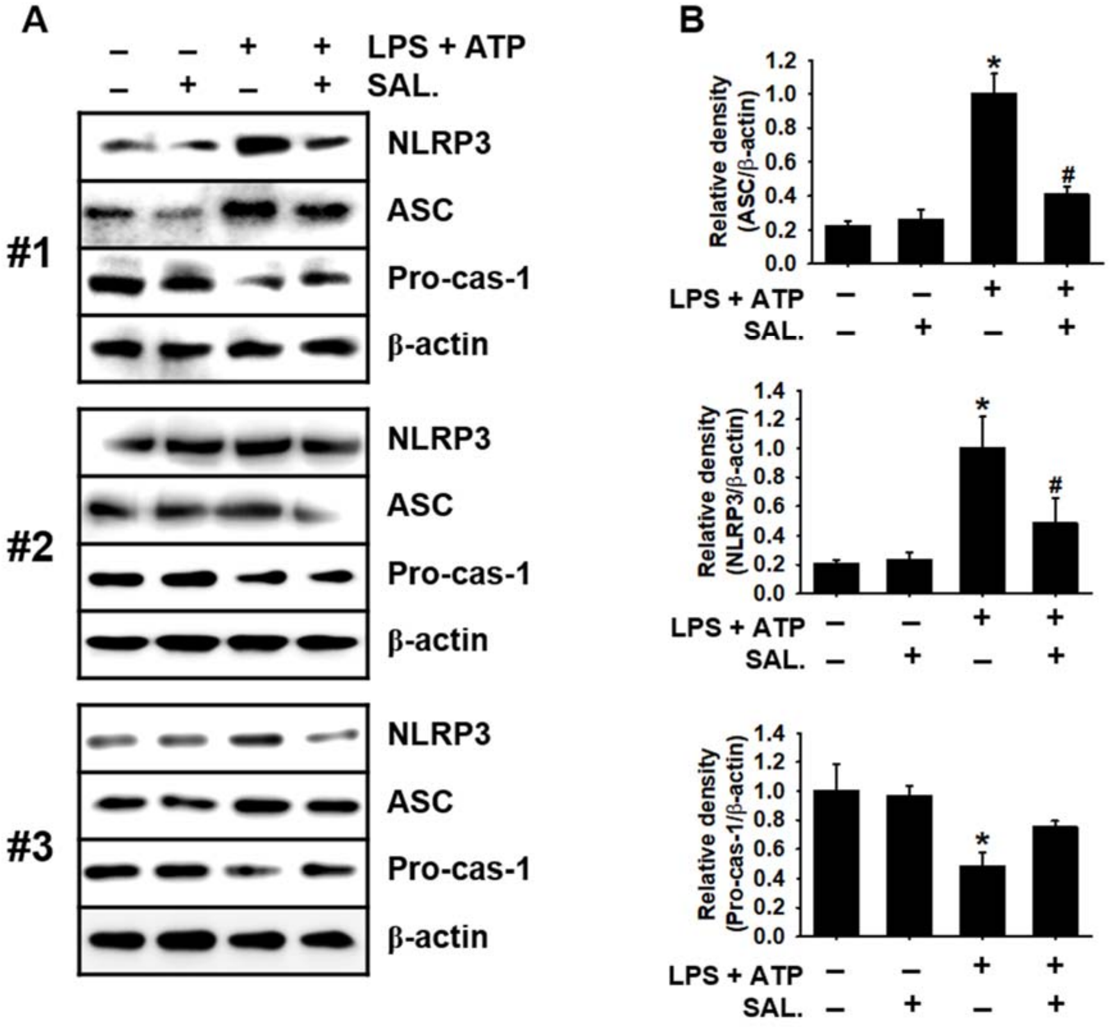

Supplementary Figure S7: Triplicate data (A) and densitometric analysis (B) of Figure 3E.

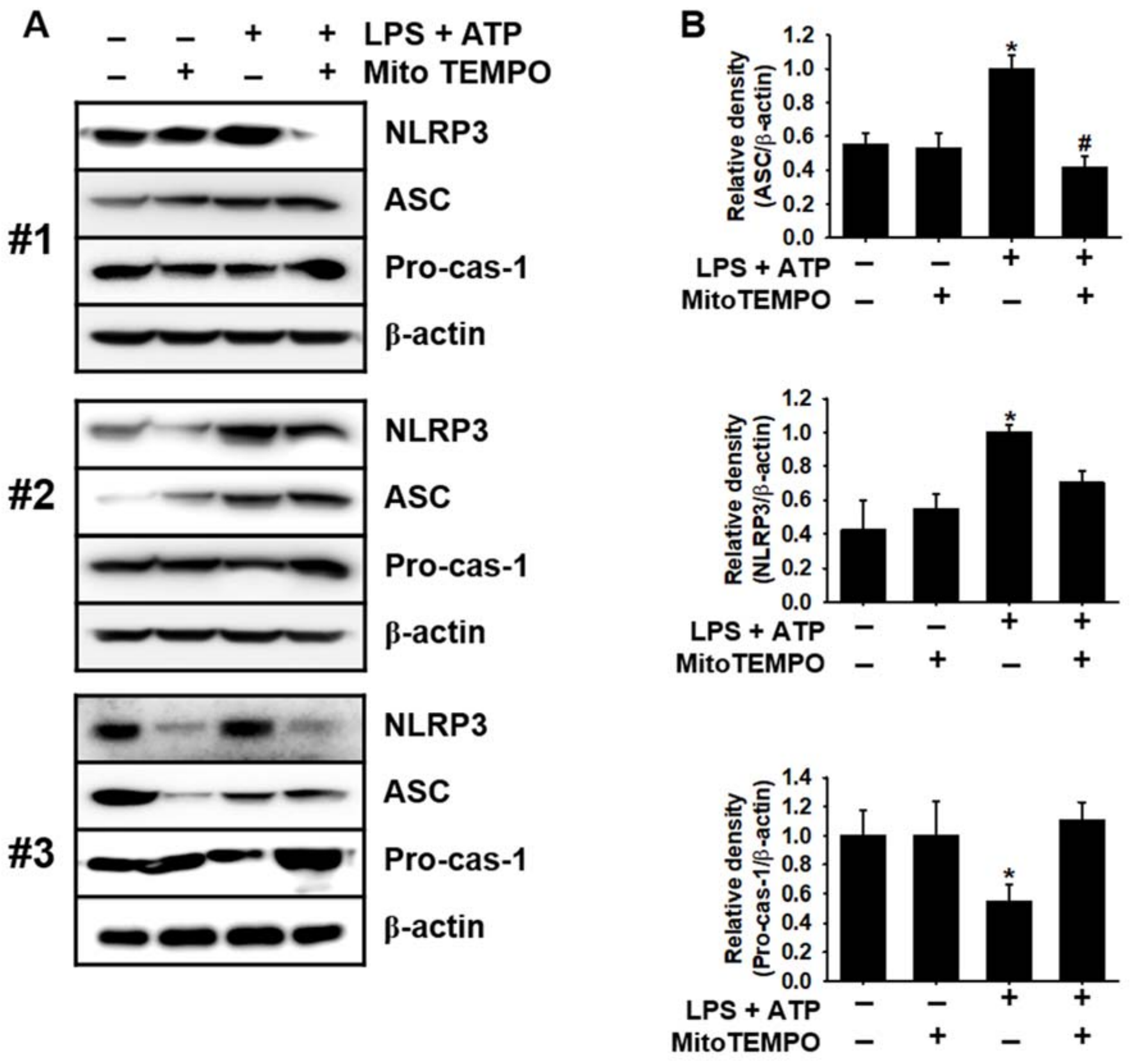

Supplementary Figure S8: Triplicate data (A) and densitometric analysis (B) of Figure 4C.

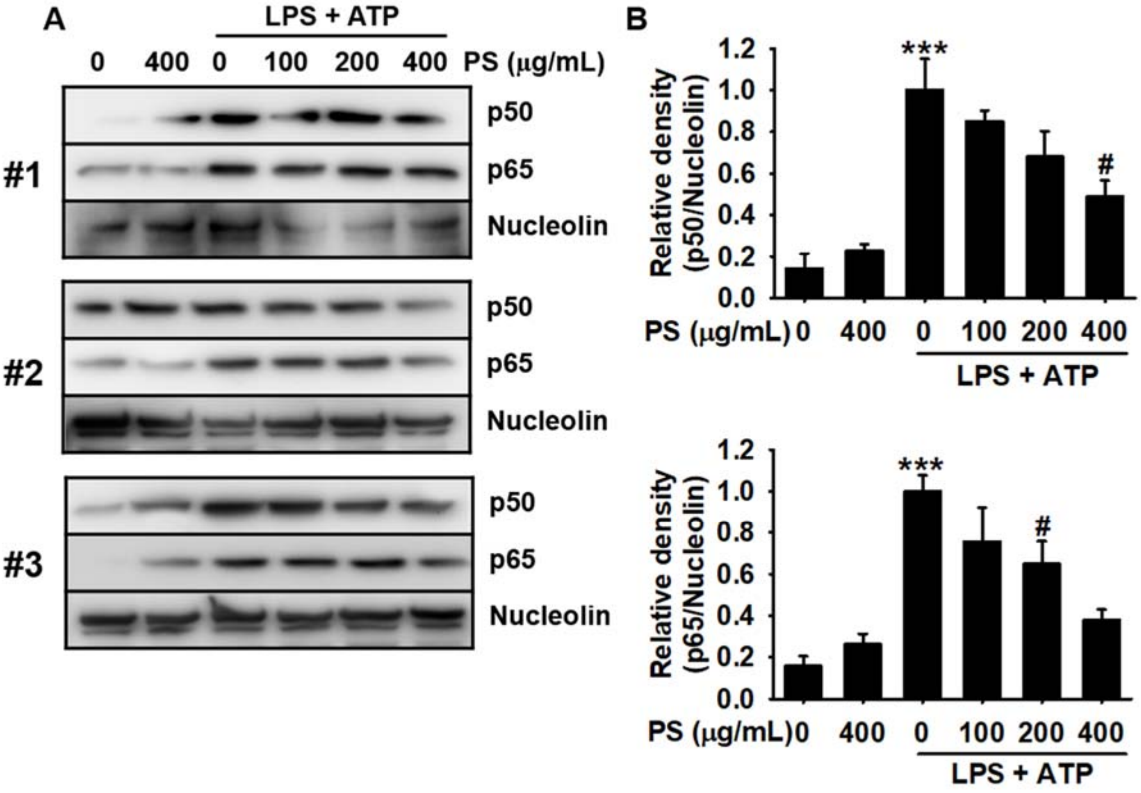

Supplementary Figure S9: Triplicate data (A) and densitometric analysis (B) of Figure 7A.

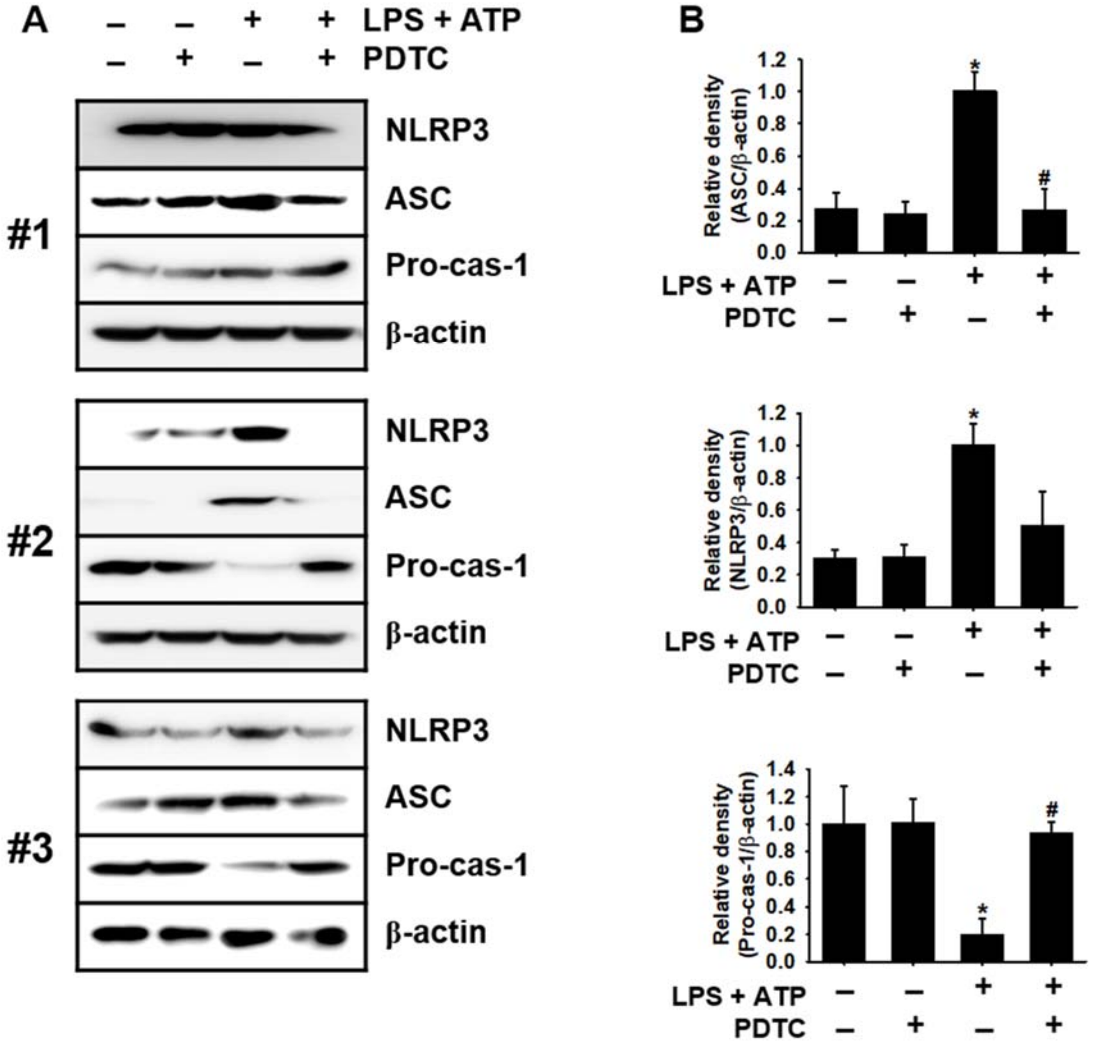

Supplementary Figure S10: Triplicate data (A) and densitometric analysis (B) of Figure 8D.
